# The impact of prophylactic azithromycin on the developing preterm gut microbiome and resistome

**DOI:** 10.1080/19490976.2026.2748147

**Published:** 2026-10-15

**Authors:** Sinead Ahearn-Ford, Ali Aboklaish, Gregory R. Young, John Lowe, Andrea C. Masi, David Gallacher, Julian R. Marchesi, Janet E. Berrington, Sailesh Kotecha, Christopher J. Stewart

**Affiliations:** a Translational and Clinical Research Institute, Newcastle University, Newcastle Upon Tyne, UK; b Department of Child Health, Cardiff University School of Medicine, Cardiff, UK; c Centre For Trials Research, Cardiff University, Cardiff, UK; d Neonatal Unit, UHW, Cardiff, UK; e Department of Metabolism, Digestion and Reproduction, Imperial College London, London, UK; f Newcastle Neonatal Service, Newcastle Upon Tyne Hospitals NHS Foundation Trust, Newcastle Upon Tyne, UK

**Keywords:** Microbiome, preterm infants, early-life antibiotic exposure, broad spectrum antibiotics, probiotics, antibiotic resistance, placebo-controlled trial, bronchopulmonary dysplasia, azithromycin, infant gut microbiome

## Abstract

Early-life antibiotic exposure can profoundly impact the developing preterm gut microbiome and resistome, with short- and long-term consequences for health. In a large multi-center (*n* = 28) study of 693 preterm infants, we investigated the effects of 10 d of prophylactic intravenous azithromycin on the gut microbiome and antibiotic resistance genes (ARGs). The AZithromycin ThErapy for Chronic Lung Disease of Prematurity (AZTEC) study was a double blind, randomized, placebo-controlled trial of preterm infants (born < 30 weeks of gestation), treated from birth for 10 d with either intravenous azithromycin or placebo, and sampled longitudinally for 21 d or discharge. Using 16S rRNA gene sequencing, antibiotic susceptibility testing, and quantitative PCR, we show azithromycin reduced microbial diversity and the relative abundance of health-associated *Bifidobacterium* and increased relative abundances of pathobionts *Staphylococcus* and *Enterobacter/Klebsiella*. This early azithromycin treatment increased the number of macrolide-resistant genes, including the presence of *erm* (C), which persisted for the duration of the study. Probiotic use (at individual unit discretion) increased alpha diversity and *Bifidobacterium* relative abundance, but did not fully mitigate the effects of azithromycin receipt. Combined azithromycin and probiotics further increased the enrichment of *Enterobacter/Klebsiella* and total ARGs, including *erm*(C). These findings highlight the impacts of antibiotic and probiotic use, both important in balancing infection control issues and microbiome disruption and antimicrobial resistance.

## Introduction

Chronic lung disease of prematurity (CLD), also known as bronchopulmonary dysplasia (BPD), is one of the most prevalent complications of preterm (<37 weeks of gestation) birth. Although the pathogenesis of CLD is multifactorial,^1^ pulmonary *Ureaplasma* colonization has been implicated in its etiology.^2^ Consequently, a recent double blind, multi-center, randomized, placebo-controlled trial (AZithromycin ThErapy for Chronic Lung Disease of Prematurity; AZTEC) sought to clarify the potential of a 10-d course of azithromycin—a macrolide antibiotic with efficacy against *Ureaplasma* and anti-inflammatory properties—on survival without physiologically defined moderate or severe CLD at 36 weeks post-menstrual age.^3^ ^,^ ^4^ Despite the AZTEC trial showing no benefit from prophylactic azithromycin, questions remain regarding the impact of systemic azithromycin administration on the developing gut microbiome and emergence of antibiotic resistance genes (ARGs) in preterm infants.^4^

Playing critical roles in early immune development, protection from pathogens, nutrient absorption and metabolism, disruption of the gut microbiome during infancy may have long-lasting health consequences.^5^ Antibiotics disrupt microbiome assembly in term and preterm infants by reducing microbial diversity, disrupting commensal populations, and increasing the colonization potential of antibiotic-resistant pathobionts.^6-15^ However, specific impacts vary depending on the duration of exposure and the choice of antibiotic. Notably, most existing data are based on observational cohorts rather than randomized trials since randomized trials of antibiotic use are rare. Previous studies specifically investigating the effects of azithromycin on the gut microbiome and ARGs have largely been restricted to children in low- to middle-income countries, with no existing preterm data.^16^

To the best of our knowledge, this study represents the first interventional trial to document the impact of prophylactic azithromycin on microbial communities in preterm infants. Previous large multisite preterm microbiome studies have indicated that site^17^ and probiotic use^18^ are among the most important factors shaping the preterm gut microbiomes. Utilizing 1261 stool samples collected from 693 of 796 recruited preterm infants (<30 weeks of gestation) over the first 21 d of life from 28 recruiting sites across the UK, this study allows further exploration of the influence of co-variates including site and probiotic use on preterm gut microbiome development over the critical first weeks of life. This study aimed to 1) describe the longitudinal development of the gut microbiome during the first 21 d of life in a cohort of preterm infants at risk of developing CLD, 2) assess the impact of azithromycin treatment on gut microbiome development and ARGs, and 3) explore relationships between microbial features, resistance genes, clinical factors, and health outcomes in this vulnerable population.

## Materials and methods

### Patient cohort and study design

This analysis included 693 preterm (<30 weeks of gestation) infants enrolled in AZTEC (registration number: ISRCTN11650227).^3^ The study protocol was approved by the Wales Research Ethics Committee 2 (ref: 18/WA/0199). Written consent was obtained before enrollment. The study was conducted in accordance with the Declaration of Helsinki and approved by the institutional ethics committee. The primary objective was to investigate the effectiveness of a 10-d course of azithromycin (intravenously at 20 mg/kg daily for 3 d, then 10 mg/kg daily for 7 d) on survival without physiologically defined moderate or severe CLD at 36 weeks postmenstrual age, in comparison to placebo (sterile water). Treatment began within 72 hours of birth. Inclusion criteria included preterm infants < 30 weeks of gestation that had respiratory support (intubation, or noninvasive mechanical ventilation, including continuous positive airway pressure and high-flow nasal cannula, or a combination of these) for at least 2 continuous hours during the first 72 hours of life, and presence of an indwelling intravenous line for drug administration. Infants were excluded if they had previous exposure to another systemic macrolide antibiotic or major congenital abnormalities.

Serial stool samples were collected opportunistically from nappies until 21 d of life (DOL). Samples were stored in sterile, polystyrene universal containers, and refrigerated immediately upon collection and stored at −80 °C at the earliest opportunity. Samples were analyzed from four time points as per trial protocol: baseline (within 72 hours of birth; before treatment), and then day 5, day 10, and days 14–21. When a sample from the specific day was not available, the closest available sample was used. When duplicate samples were collected on the same day, the sample with the highest sequencing reads was chosen. Demographic data were collected at baseline and clinical follow-up data were collected at 36 weeks postmenstrual age

### 16S rRNA gene sequencing and taxonomic profiling

DNA was extracted from all collected stool samples (*n* = 1797) using ~0.1 g of sample with the DNeasy PowerLyzer PowerSoil Kit (QIAGEN). An extended bead-beating step was performed by vortexing the samples using a vortex adapter at maximum speed for 20 minutes. DNA was incubated in 70 μL of solution C6 for five minutes at room temperature before the final elution to maximize yields, and eluted DNA was immediately stored at −80 °C. Two negative controls (containing no biological sample) were extracted with every batch of 96 samples (*n* = 37).

Sequencing of the V3-V4 region of the 16S rRNA gene was performed using primers 341F (CCTACGGGNGGCWGCAG) and 805 R (GACTACHVGGGTATCTAATCC) on the Illumina MiSeq platform, generating paired-end reads of 2 × 300 base pairs, by the Integrated Microbiome Resource (Dalhousie University, Halifax, Canada). The protocol follows the method originally described by Comeau et al.^19^ and updated by Comeau and Kwawukume.^20^ All raw paired-end sequencing data were imported into QIIME 2^21^ and sequence quality filters were applied with DADA2.^22^ To account for low base-call quality at terminal ends of sequencing reads (25% Phred < Q30), the first 15 and last 40 nucleotides were trimmed from forward reads, while the first 10 and last 80 nucleotides were trimmed from reverse reads. Remaining reads with low-quality base calls were removed. Representative sequences for each amplicon sequence variant (ASV) were identified by dereplicating all reads passing quality control. ASVs were clustered based on 97% similarity with VSEARCH^23^ before assigning taxonomy to the SILVA database (v138).^24^ All samples were rarefied to 1000 reads using the vegan package version 2.6.4,^25^ retaining 1261 samples for analysis. Good's Coverage between rarefied and unrarefied data which showed 1000 reads captured most of the diversity within these samples (raw median 0.9997 (IQR 0.9958–0.9999); rarified median 0.9970 (IQR 0.9950–0.9980)).

### Quantitative PCR

Quantitative polymerase chain reaction (qPCR) was performed on 793 samples and targeted six macrolide resistance genes, which account for >90% of bacterial macrolide resistance (Supplementary Table 1). Macrolide-resistance genes (*n* = 6) were detected using a published multiplex qPCR assay.^26^ Briefly, qPCR reactions comprised: 5 μL 2× iTaq Universal Probes Supermix (Bio-Rad Laboratories), 0.5 μL (10 μM) each forward and reverse primers of the six macrolide-resistance genes, 0.2 μL (10 μM) each of the six hydrolysis probes, and molecular-grade water to make up a final volume of 10 μL. All qPCR reactions were performed in duplicate on a CFX96 Touch Real-Time PCR detection system (Bio-Rad Laboratories Ltd), using the cycling conditions: initial activation and denaturation 95 °C for 5 minutes, followed by 40 cycles of 95 °C for 15  seconds and 60 °C for 30 seconds. A standard curve of 10-fold dilutions (10^7^–10^2^ copy number) of a plasmid DNA containing primer and probe target sequences of the six genes (GenScript, USA) was generated for each run of the assay.

### Antibiotic susceptibility testing of *Bifidobacterium* strains

Antibiotic susceptibility was investigated for eight *Bifidobacterium* isolates with seven of eight cultured from preterm stool samples: *Bifidobacterium breve* (PP1, VK68, AM76, JD156), *Bifidobacterium infantis* (VK53, AM7, LB1 (cultured from Labinic probiotic)), and *Bifidobacterium bifidum* (CS27), alongside two control type strains: *Escherichia coli* (DSM 1103) and *Staphylococcus aureus* (DSM 2569). *Bifidobacterium* strains were inoculated in De Man–Rogosa–Sharpe (MRS) broth with L-cysteine, and control strains were transferred to brain heart infusion (BHI) broth and incubated anaerobically and aerobically at 37 °C, respectively. Minimum inhibitory concentrations (MICs) were determined using the Epsilometer test (E-test) method.^27^ Optical density (OD600nm) of the cultures was adjusted to obtain a cell count of ~6 × 10^8^ CFU/mL (equivalent to 0.8 McFarland standard). Cultures were spread on the surface of Brucella agar supplemented with 5% defibrinated sheep's blood (Thermo Scientific™ Oxoid™) in three directions using cotton swabs. E-test strips (Biomérieux) for azithromycin, gentamicin, and benzylpenicillin were applied to inoculated plates according to the manufacturer's instructions. Plates were incubated at 37 °C for 48 hours for *Bifidobacterium* strains (anaerobically) and 24 hours for control strains (aerobically), before MICs were determined. Reference values from the European Committee on Antimicrobial Susceptibility Testing (EUCAST) quality control^28^ and breakpoint tables^29^ were used to interpret MIC results.

### Statistical analysis

All statistical analyzes were performed in R software version 4.3.1^30^ on rarefied samples. Unless stated otherwise, all visualizations were produced using the ggplot2 package version 3.4.3.^31^ *P* values < 0.05 were deemed significant.

#### Clinical demographic and outcome data

Data were collected for AZTEC onto CRFs at recruitment and daily. Except for DOL, season of sample collection, and probiotic exposure at the time of the sample, all other clinical variables were fixed from birth. Binary variables were included only if they were represented by at least 5% within either category at each time point (except in the case of probiotic exposure, which did not occur for any infants until the day 5 time point). Continuous variables were presented as median (interquartile range; IQR), and included sample DOL, gestational age at birth (weeks+days) and birth weight (grams). Categorical variables were shown as proportions, and included treatment group (azithromycin/placebo), sex (male/female), delivery mode (vaginal/cesarean), UK-defined season of sample collection (spring/summer/autumn/winter), cause of preterm birth (classified as preterm premature rupture of membranes (PPROM) (yes/no), preterm labor without PPROM (yes/no), obstetric medical intervention for fetal reasons (yes/no)), pregnancy plurality (single/multiple), exposure to antenatal antibiotics within 5 d of delivery (yes/no), maternal ethnicity (white British/other), site (included 28 recruiting sites), and infant exposure to probiotics ever (yes/no). Of infants that received probiotics, 84% received Labinic (*Lactobacillus acidophilus*, *Bifidobacterium bifidum*, and *Bifidobacterium infantis*), 4% received ProPrems (*Bifidobacterium infantis*, *Bifidobacterium lactis*, and *Streptococcus thermophilus*), and 12% received Infloran (*Lactobacillus acidophilus* and *Bifidobacterium bifidum*). Differences between treatment groups were calculated using Fisher's or Chi-Square test (dependent on cell counts) for categorical variables, or Wilcoxon rank sum test for continuous variables.

Binary (yes/no) clinical outcomes included mortality at discharge from the neonatal unit, physiologically defined moderate or severe CLD at 36 weeks postmenstrual age, nosocomial infections (positive blood culture, cerebrospinal fluid culture, or both, or antibiotic treatment for ≥5 d), severe intraventricular hemorrhage (IVH, grade III or IV), necrotizing enterocolitis (NEC; Bell stage ≥ II), treatment for retinopathy of prematurity (ROP), and treatment for patent ductus arteriosus (PDA). Differences in outcomes between treatment groups were calculated using Chi-Square tests. Associations between clinical outcomes and alpha diversity were tested by addition of each outcome to mixed-effects linear regression models; fixed effects included those in multivariate alpha diversity analyzes: treatment group, sample DOL, mode of delivery, gestational age, sampling season, preterm labor with PPROM, antenatal antibiotics, medically indicated preterm birth, and infant probiotics. Patient identifier and site were included as random effects. Global *p*-values for variables from the final fitted models were obtained by type II analysis of variance in the car package. Associations with taxonomy were tested by addition of each outcome to MaAsLin2^32^ global models; fixed effects included those in other MaAsLin2 analyzes: treatment group, sample DOL, mode of delivery, gestational age, sampling season, preterm labor with PPROM, medically indicated preterm birth, antenatal antibiotics, and infant probiotics, and random effects included patient identifier, site, and twin group.

#### Technical variables

Four technical variables were investigated to assess their impact on microbiome data: extraction batch (categorical), sequencing plate (categorical), sequencing run (categorical), and date of sample collection (continuous; calculated as days from collection of the first study sample). None of the technical variables investigated had any significant impact on alpha or beta diversity, so technical variables were not considered in any further analyzes (all *p*-values > 0.05).

#### Alpha diversity

Alpha diversity based on number of OTUs (richness) and Shannon diversity (richness and evenness) were computed using the vegan package.^25^ Longitudinal alpha diversity was modeled using locally estimated scatterplot smoothing (LOESS) regression and plotted with 95% confidence intervals (CIs). Spearman's correlation was used to assess alpha diversity relationships amongst related and unrelated infant pairs at each time point, with FDR correction.^33^ Univariable associations with treatment group, clinical variables, and technical variables were assessed using Spearman's rank correlation (for continuous variables) or Wilcoxon rank sum/Kruskal Wallis (for categorical variables), with FDR correction. Effect sizes were calculated for categorical clinical variables using functions wilcox_effsize and kruskal_effsize from the rstatix package version 0.7.2.^34^ Mixed effects linear regression models were fitted for box-cox-transformed bacterial richness and Shannon diversity using lme4 package version 1.1.35.5^35^ and MASS package version 7.3.60.0.1.^36^ The models were adjusted for factors reported in the literature to be associated with infant microbiomes and those that were significantly associated with alpha or beta diversity at any time point; fixed effects included treatment group, sample DOL, mode of delivery, gestational age, sampling season, preterm labor with PPROM, antenatal antibiotics, medically indicated preterm birth, and infant probiotics. Patient identifier and site were included as random effects. Birth weight was excluded from the models due to collinearity with gestational age (Pearson's R > 0.75 at all time points). Variance inflation factor was calculated for the models using the car package version 3.1.2,^37^ with a conservative maximum cut-off of <2. Global *p*-values for variables from the final fitted models were obtained by type II analysis of variance in the car package. Post hoc analysis was performed by pairwise comparisons (Tukey's HSD method) from the emmeans package version 1.10.0.^38^

#### Beta diversity and ordination

Beta diversity was analyzed based on weighted Bray‒Curtis and unweighted Jaccard distance metrics from the vegan package^25^ and compared using Wilcoxon rank-sum tests with FDR correction.^33^ PERMANOVA was performed with 1000 permutations using the “adonis2” function of the vegan package^25^ with FDR correction.^33^ Significant findings were further assessed for homogeneity of variances using Permutational Multivariate Analysis of Dispersion (PERMDISP) via the “betadisper” function in the vegan package. If both PERMANOVA and PERMDISP results were significant, the Wd* method^39^ and subsetting procedures were applied. Due to the impact of twins on beta diversity, analyzes were repeated on reduced datasets including only one randomly selected twin pair/triplet set, ensuring robustness of significant findings for clinical variables.

Ordinations were conducted using Non-metric Multidimensional Scaling (NMDS) by the “metaMDS” function of the vegan package.^25^ Stress values were calculated to assess goodness-of-fit and Shepard plots were generated to visually inspect the correspondence between original dissimilarities and distances in the NMDS solution. Ordinations were permissible with stress values <0.15.

#### Taxonomy

Taxonomic compositions of stool samples were explored at the phylum and genus level and expressed as relative abundances per sample. Longitudinal relative abundance data were modeled using locally estimated scatterplot smoothing (LOESS) regression and plotted with 95% CIs. MaAsLin2 models included factors previously reported to be associated with infant microbiomes and those that were significantly associated with alpha or beta diversity at any time point. Site and twin group were included as random effects when relevant within time point models, and patient identifier was additionally included as a random effect in the global models. The arcsin square root transformation was performed on relative abundance data and default MaAsLin2 parameters were used. All *p*-values were adjusted for multiple comparisons using FDR adjustment (Benjamini–Hochberg procedure) and default q-values of <0.25 were deemed significant. To assess the combined impact of treatment group and probiotic exposure, a composite variable integrating both factors was created and used in place of the individual variables in the MaAsLin2 time point models.

#### Antibiotic resistance genes

A total of six ARGs associated with macrolide resistance were investigated (Supplementary Table 1). ARGs were explored based on the presence or absence of each gene. Relationships between each ARG and relative abundances of the top 15 genera and top 4 phyla were compared using Wilcoxon tests followed by FDR adjustment.^33^ Wilcoxon effect size was calculated to assess the strength of relationships using rstatix package.^34^ To understand the relationship between DOL and the presence/absence of ARGs, mixed-effects binomial logistic regression models with logit link were fit for each ARG using “glmer” function from lme4 package,^35^ including patient identifier as a random effect. To observe relationships with total number of ARGS, cumulative link mixed models were fit using “clmm” function from package ordinal version 2023.12.4,^40^ including patient identifier as a random effect.

## Results

### Cohort samples and characteristics

In total, 1261 stool samples from 693 preterm infants (<30 weeks' gestation) were included in the analysis (Supplementary Figure 1A). Low sequencing depth (<1k reads) was more common in samples collected at an earlier age, resulting in the proportion of samples passing rarefaction increasing over time (Supplementary Figure 1B). Consequently, only a small proportion of infants (4.5%) from the overall cohort were included at the baseline time point (Supplementary Figure 1C). Infants contributed a median of 2 samples (IQR: 1–2), with only three infants having samples available at all four time points. Demographic information and clinical characteristics of the cohort are presented in Supplementary Table 2. Study infants had a median gestational age at birth of 27 + 1 weeks (IQR: 25 + 3 to 28 + 5) and birth weight of 903 g (IQR: 720 to 1140).

### Overview of temporal gut microbiome development over the first 21 d and associations with clinical co-variates

We first sought to perform a descriptive analysis of this large multi-center cohort and elucidate the factors driving gut microbiome that could inform confounder selection for subsequent analysis. Bacterial richness declined slightly over the first 10 d, before recovering and increasing gradually thereafter, while Shannon diversity increased steadily from birth (Supplementary Figure 2A). Related infants (*n* = 69 pairs of twins and *n* = 6 sets of triplets) displayed significantly increased similarity in microbial composition compared to matched unrelated infant pairs at all time points by both Bray‒Curtis and Jaccard distances (all *p* < 0.05; Supplementary Figure 3).

Analysis of alpha diversity showed older DOL, vaginal birth, probiotic exposure, and samples collected during summer were associated with increased bacterial richness (*p* values < 0.05; Table 1). Shannon diversity was also higher in older DOL, probiotic-exposed infants, and during the summer season (*p*-values < 0.05; Table 1). Beta diversity was significantly associated with gestational age, birth weight, delivery mode, preterm labor with preterm premature rupture of membranes (PPROM), medically indicated preterm birth, antenatal antibiotics, infant exposure to probiotics, and site, at various time points (*p* values < 0.05; Figure 1). Among these, recruiting site explained the largest proportion of variance thus was subsequently included in multivariate analyzes as a random effect. No significant associations were detected at baseline, likely reflecting lower sampling numbers at this time point.

**Table 1.** Linear mixed effects models of treatment group and clinical co-variates on alpha diversity.

Variable	Richness		Shannon diversity	
	Chisq	*p* value	Chisq	*p* value
Treatment group	5.8566	0.0155	14.5159	0.0001
Day of life	26.8549	2.19 × 10^−7^	85.1997	<2.2 × 10^−16^
Delivery mode	10.8563	0.001	1.0663	0.3018
Gestational age	0.2501	0.617	0.0018	0.9658
Preterm labor with PPROM	0.7609	0.383	0.0004	0.984
Medically indicated preterm	1.6632	0.1972	0.0032	0.9547
**birth**				
Antenatal antibiotics	1.6550	0.1983	2.6854	0.1013
Infant probiotics	9.1434	0.0025	4.4954	0.034
Season	12.1707	0.0068	8.9663	0.0297

PPROM, preterm premature rupture of membranes.

**Figure 1.**
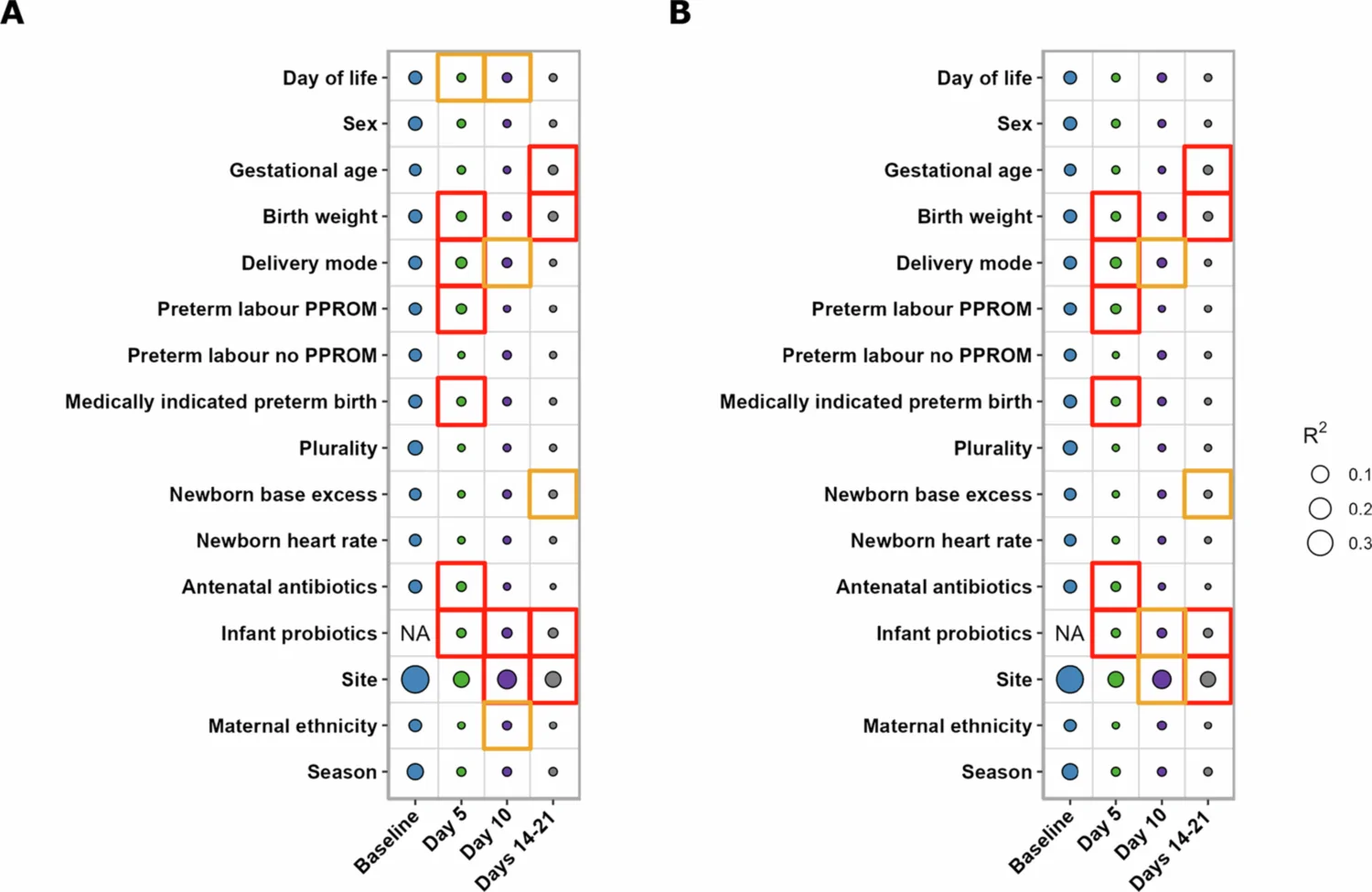
Significance and explained variance of clinical co-variates at given time points. Bubbles represent the amount of variance (R^2^) explained by each variable based on A) Bray-Curtis and B) Jaccard distance matrices. Bubbles are colored according to time point (baseline, day 5, day 10, or days 14–21). R^2^ and *p* values are from “adonis2”; red boxes indicate FDR-corrected *p*-value <  0.05 and orange boxes indicate results trending towards significance (<0.1). PPROM, preterm premature rupture of membranes.

Considering taxonomic relative abundance, later DOL and higher gestational age at birth were significantly associated with increased relative abundances of Pseudomonadota and decreased Bacillota phyla, corresponding to increased *Enterobacter/Klebsiella* and *Escherichia/Shigella*, and decreased *Staphylococcus* genera (q values < 0.25; Supplementary Figure 2B and 2C; Supplementary Table 3). Later DOL also had higher relative abundances of *Bifidobacterium* and *Enterococcus* (q values < 0.25). In comparison to those delivered by cesarean section, vaginally born infants had higher relative abundances of Pseudomonadota and lower Bacillota phyla, as well as increased *Escherichia/Shigella* and decreased *Staphylococcus* genera (q values < 0.01; Supplementary Table 3).

### Azithromycin treatment altered gut bacterial diversity and composition

Having defined the main factors impacting gut microbiome development over the sampling period, we next sought to determine the impact of azithromycin. In comparison to placebo, bacterial richness in azithromycin-treated infants was significantly lower from day 10 time point (Figure 2A), and Shannon diversity was significantly lower from day 5 (*p* values < 0.05; Figure 2B). Bray-Curtis beta-diversity analysis further showed overall microbial composition was significantly different between azithromycin and placebo infants at day 10 and days 14–21 time points (*p* values < 0.05; Figure 2C).

**Figure 2.**
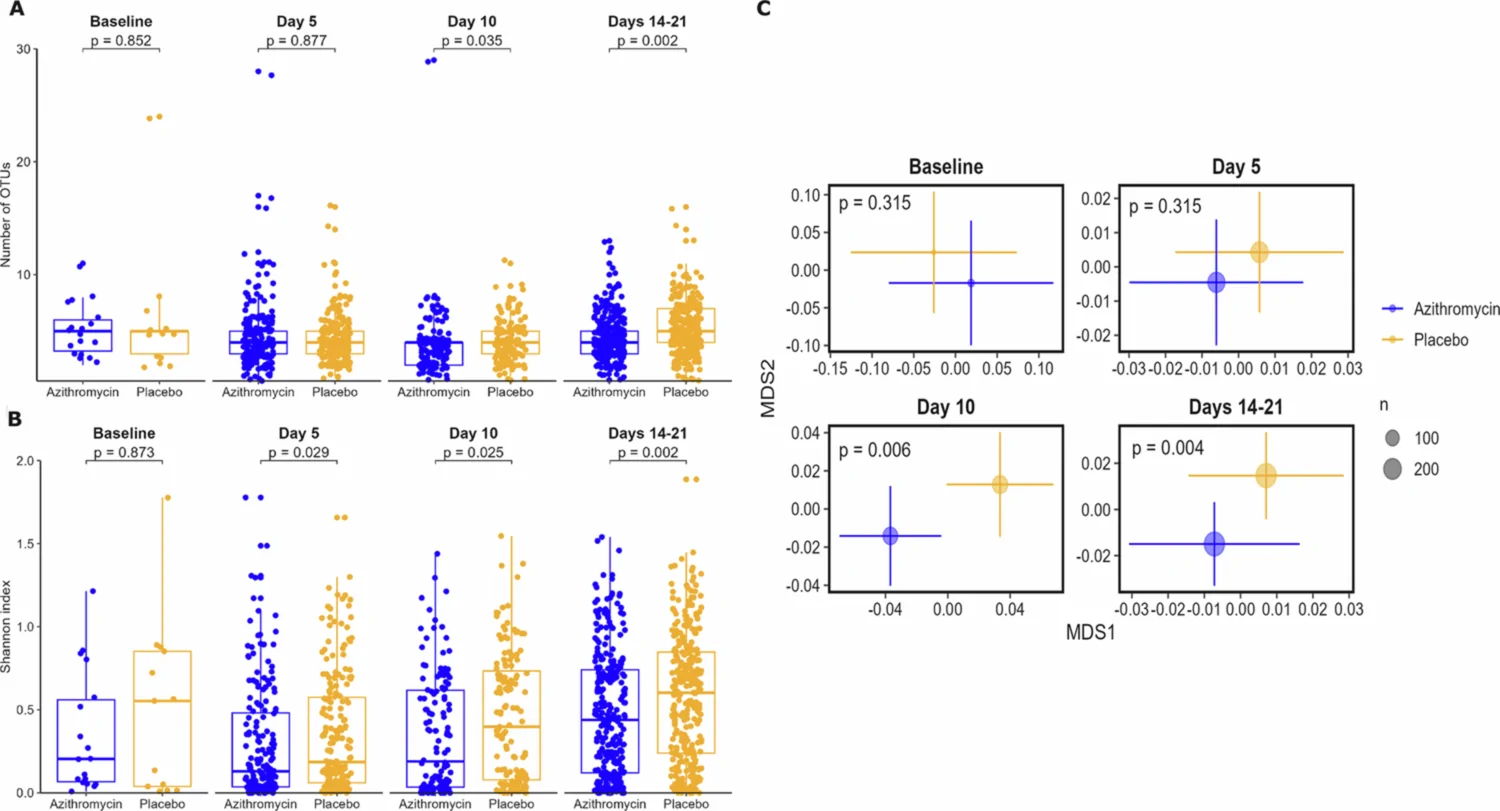
Alpha and beta diversity of preterm infants treated with azithromycin or placebo at given time points. A) Number of OTUs and B) Shannon diversity of preterm infants treated with azithromycin or placebo. Boxes are colored according to treatment group and show median (center line) and interquartile ranges (IQR) (box limits); whiskers extend ± 1.5∗IQR from box's quartile. Points outside the whiskers represent outliers. *P* values are from FDR-adjusted Wilcoxon rank-sum tests. C) NMDS plots are based on ordination of Bray–Curtis distance matrices. Plots show the mean centroids for each group and centroid size is based on the number (*n*) of samples, with bars representing the ±95% CI. *P* values (FDR-corrected) on plot faces are from “adonis2”.

After adjusting for co-variates, significantly higher Pseudomonadota and lower Actinomycetota relative abundances were observed at the 14–21 d time point in azithromycin-treated infants (q values < 0.05; Supplementary Figure 4A and Supplementary Table 4). At the genus level, *Bifidobacterium* relative abundance was consistently lower in azithromycin-treated infants from day 5 (q values < 0.25; Figure 3A, Supplementary Table 4 and Supplementary Figure 4B). *Bifidobacterium* was the third most abundant genus at days 14–21 in placebo-treated infants, but sixth most abundant in azithromycin-treated infants. Additionally, azithromycin-treated infants had lower relative abundance of *Enterococcus* at days 5 and 10 time points, coupled to higher relative abundances of *Staphylococcus* at days 5 and 10 and *Enterobacter/Klebsiella* at days 14–21 (q values < 0.25; Supplementary Figure 4B and Supplementary Table 4). We next explored prevalences of genera within each treatment group, finding the proportion of infants with detectable *Bifidobacterium* increased between adjacent time points in placebo-treated infants, but declined after the day 5 time point in azithromycin-treated infants, becoming significantly lower by days 14–21 (*p*-value < 0.001; Figure 3B). The prevalence of *Clostridium sensu stricto* also increased over time in placebo-treated infants, but remained relatively low in azithromycin-treated infants, and was significantly lower at days 14–21 in those given azithromycin (*p*-value < 0.001).

**Figure 3.**
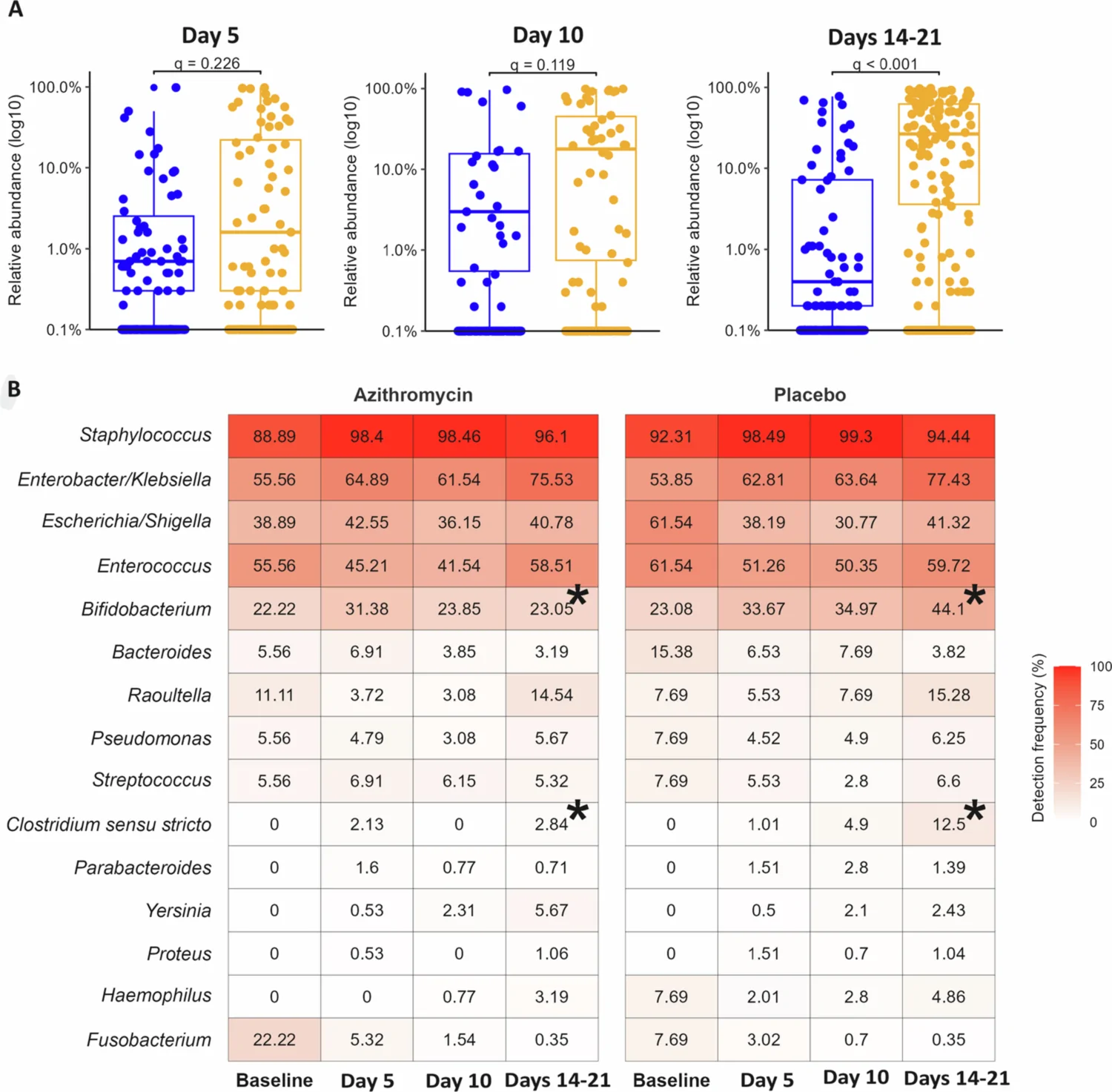
Longitudinal changes in genus-level prevalence in the gut microbiomes of preterm infants treated with azithromycin or placebo. A) Boxplots showing the relative abundance of *Bifidobacterium* at day 5, day 10, and days 14–21. Boxes are colored according to treatment group and show median (centre line) and interquartile ranges (IQR) (box limits); whiskers extend ± 1.5∗IQR from box's quartile. Points outside the whiskers represent outliers. Q values (FDR-adjusted *p-*values) shown are from MaAsLin2. B) Heatmaps showing the percentage of patients within each treatment group in which the top 15 genera were identified at baseline, day 5, day 10, and days 14–21. Significant differences between groups are from Chi-squared tests and are indicated with an asterisk (FDR corrected *p* values < 0.001).

### *Bifidobacterium* strains exhibited variable susceptibility to azithromycin in vitro

Given the observed reduction in the relative abundance and prevalence of *Bifidobacterium*, *in vitro* E-tests were conducted to assess the susceptibility of *Bifidobacterium* strains to azithromycin as well as benzylpenicillin and gentamicin owing to their common use in preterm infants (Table 2). MIC values for azithromycin against *Bifidobacterium* strains ranged from 0.064 to 1.5 μg/mL, with *Bifidobacterium breve* isolates showing the highest sensitivity to azithromycin (median 0.5 μg/mL; IQR 0.23–0.5 μg/mL). While no established azithromycin breakpoint values exist for *Bifidobacterium* or other anaerobic bacteria, one study providing general MIC data for azithromycin in Gram-positive anaerobes indicates MIC50 values of 1 μg/mL and MIC90 values of 256 μg/mL.^41^ On this basis, the majority of *Bifidobacterium* strains tested here, excluding *Bifidobacterium infantis* (VK53; MIC 1.5 μg/mL), had lower resistance and potentially higher susceptibility to azithromycin than other Gram-positive anaerobes like *Lactobacillus* spp. MICs for benzylpenicillin were also low, but had a narrower range, with values from 0.064 to 0.38 μg/mL. Gentamicin had the lowest inhibitory activity and the broadest MIC range, with values between 32 and 96 μg/mL.

**Table 2.** Minimum inhibitory concentration values from E-tests, given as μg/Ml.

Strain	Azithromycin	Benzylpenicillin	Gentamicin
*Staphylococcus aureus*	2 (QC)	0.75 (QC)	1 (QC)
*Escherichia coli*	3 (N/A)	0.75 (N/A)	1 (QC)
*Bifidobacterium breve* (VK68)	0.064	0.38	32
*Bifidobacterium breve* (AM76)	0.5	0.25	64
*Bifidobacterium infantis* (VK53)	1.5	X	32
*Bifidobacterium infantis* (AM7)	0.5	0.25	48
*Bifidobacterium bifidum* (CS27)	0.25	0.064	96

QC, control strain was within the given EUCAST quality control range; N/A, no available EUCAST quality control data for control strain; X, result was not clear.

### Probiotics influenced the preterm gut microbiome but could not perturb the effects of azithromycin treatment

A total of 62% of recruiting sites used probiotics, all of which contained at least one *Bifidobacterium* species (Supplementary Table 2). Exposure to probiotics had the most significant and consistent impact on bacterial profiles (Figure 1). Infants receiving probiotics exhibited increased alpha diversity, increased relative abundances of Actinomycetota phylum and *Bifidobacterium* genus, and decreased relative abundances of Bacillota phylum and *Escherichia/Shigella* genus (q values < 0.25; Supplementary Figure 5). *Lactobacillus* was present in the probiotic formulations used by 96% of probiotic-exposed infants in this study. However, this taxon was not detected in the 16S rRNA gene profiles generated using the QIIME2 pipeline. Preliminary analyzes using Kraken2^42^ on the same 16S rRNA gene reads identified *Lactobacillus* at low relative abundance (0.06% in azithromycin-treated infants and 0.3% in placebo-treated infants). MaAsLin2 re-analysis using Kraken2-derived taxonomic profiles did not reveal any statistically significant associations between probiotic use and *Lactobacillus* relative abundance.

Because exposure to probiotics increased alpha diversity and *Bifidobacterium* relative abundance—two factors negatively impacted by azithromycin treatment—we next sought to investigate the extent to which azithromycin treatment effects could be mitigated by probiotic use. Four subgroups were created based on treatment status and probiotic exposure: (1) azithromycin-treated infants with probiotic exposure (AZM+Pro+), (2) azithromycin-treated infants without probiotic exposure (AZM+Pro), (3) placebo-treated infants with probiotic exposure (AZM- Pro+), and (4) placebo-treated infants without probiotic exposure (AZM- Pro-). The relative abundance of *Bifidobacterium* was significantly higher in AZM- Pro+infants compared to all other subgroups at all time points (q values < 0.25; Figure 4C and Supplementary Table 5). Focusing on the differences between probiotic-exposed infants at the days 14–21 time point, compared to AZM+Pro+infants, AZM- Pro+infants showed significantly higher alpha diversity (richness *p* < 0.01; Shannon *p* < 0.05; Figure 4A and B) and *Bifidobacterium* relative abundance (q value < 0.001; Figure 4C). This indicates that probiotics did not prevent the reduced alpha diversity and failed to counteract the reduction in *Bifidobacterium* caused by azithromycin treatment.

**Figure 4.**
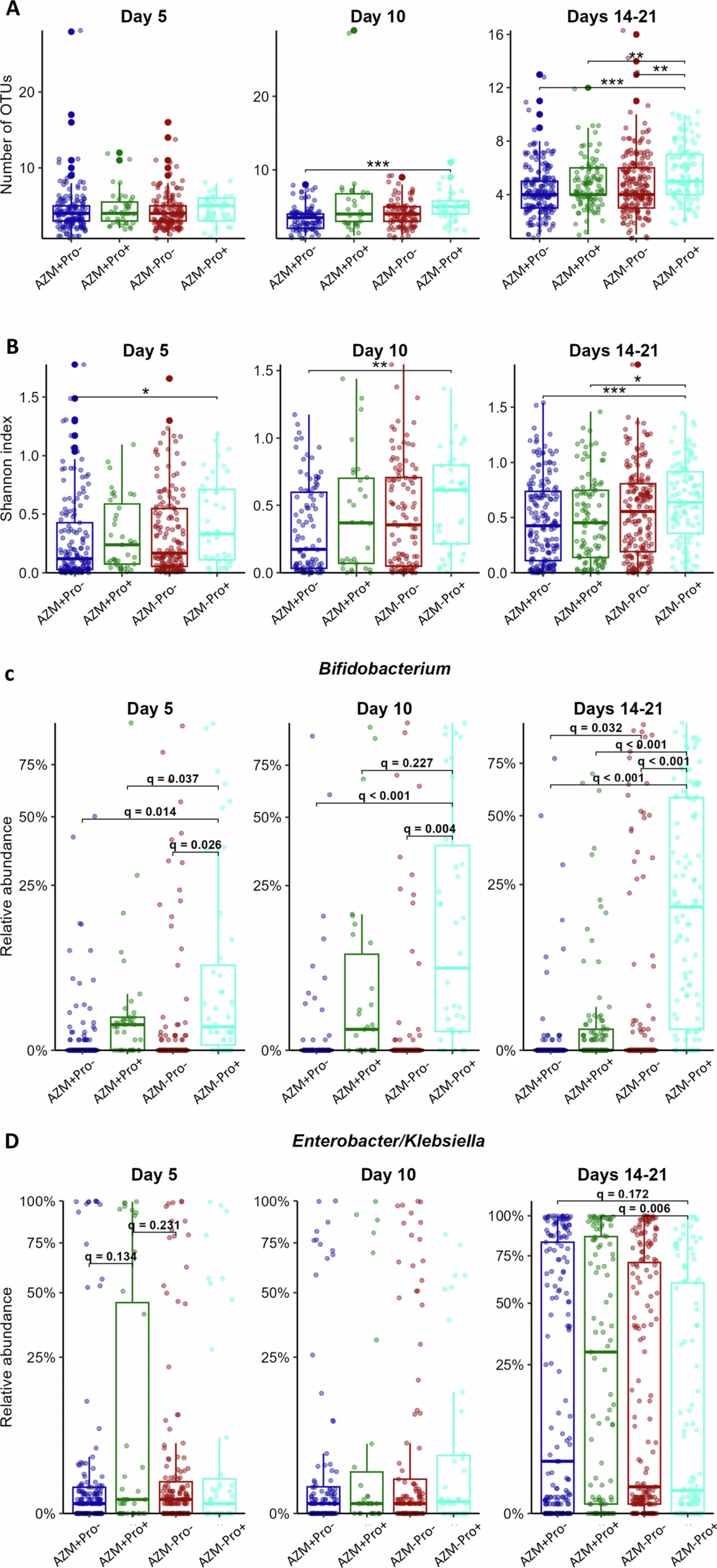
Probiotics did not mitigate the effects of azithromycin treatment on the preterm gut microbiome. Boxplots show A) the number of OTUs, B) Shannon diversity, and C,D) the relative abundance of C) *Bifidobacterium* and D) *Enterobacter/Klebsiella* in infants treated with azithromycin (with and without probiotics, AZM+Pro+and AZM+Pro-, respectively) or placebo (with and without probiotics, AZM- Pro+and AZM- Pro-, respectively) at given time points. Boxes are colored according to treatment/probiotic group and show median (center line) and interquartile ranges (IQR) (box limits); whiskers extend ± 1.5∗IQR from box's quartile. Points outside the whiskers represent outliers. In A) and B), following a significant Kruskal‒Wallis test, *p*-values shown are based on the Dunn test with Holm‒Bonferroni adjustment for multiple comparisons; * indicates *p*-value < 0.05, ** indicates *p*-value < 0.01, *** indicates *p*-value < 0.001. Q values (FDR-adjusted *p*-values) in C) are from MaAsLin2, after adjusting for confounders.

### Temporal development of preterm macrolide resistance genes and their relationships with gut taxa

We next employed qPCR to assess the presence of macrolide resistance genes (*n* = 793 stool samples) (Supplementary Table 1). The prevalence of each ARG was significantly positively associated with increasing DOL (*p*-values < 0.05; Figure 5A). *msr*(A) was the most prevalent and showed the largest increase in odds per unit increase in DOL (30%), while *mef*(A/E) was the least common with the smallest increase in odds (14%). The total number of ARGs detected within a sample also demonstrated a significant positive association with DOL (estimate = 0.197, SE = 0.016, z = 12.52, *p* < 0.001).

**Figure 5.**
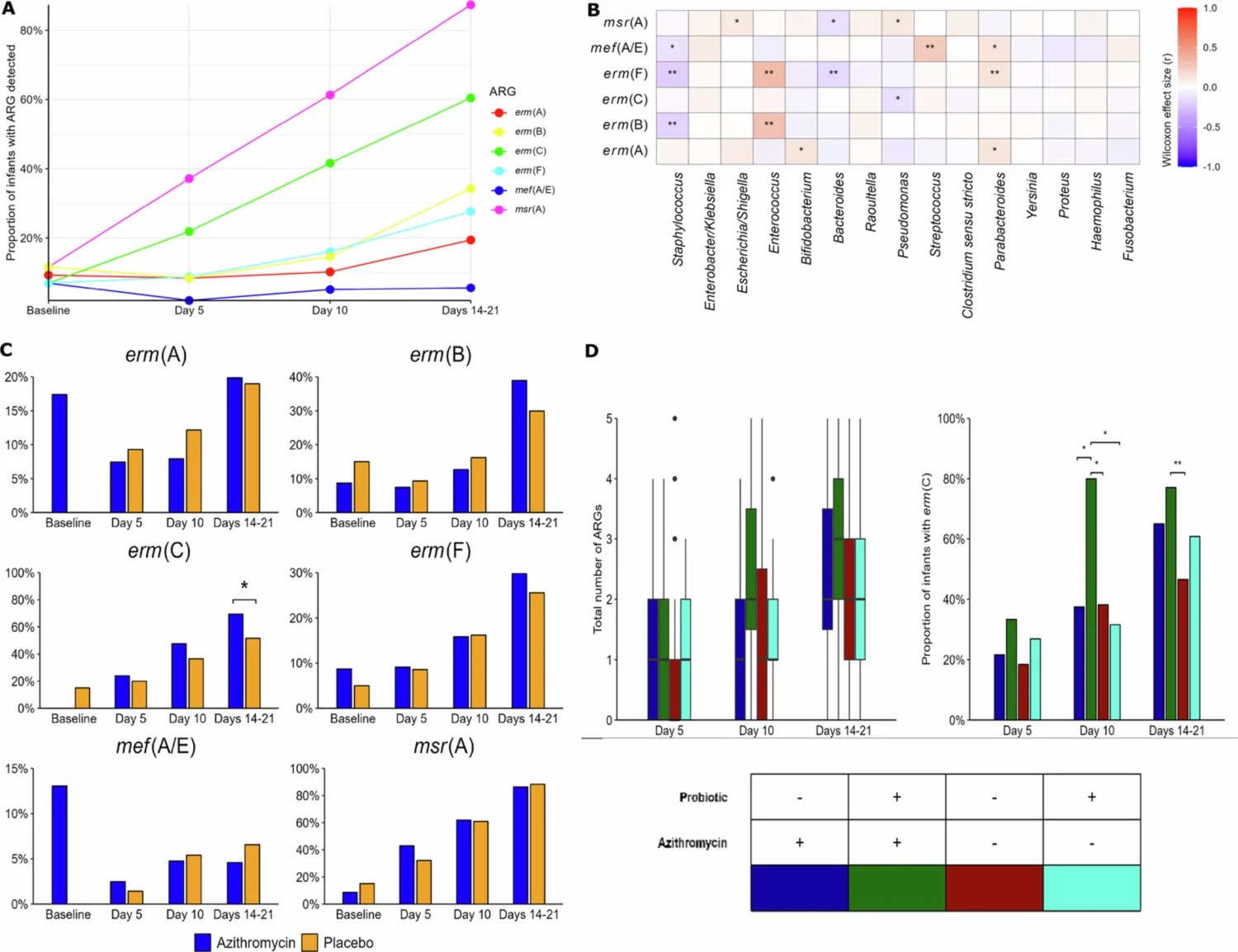
Longitudinal development of antibiotic resistance genes (ARGs) and their association with azithromycin and probiotic treatments. A) Line plots show the proportion of infants with each antibiotic resistance gene (ARG) present at given time points. B) Associations between antibiotic resistance genes and the relative abundance of the top 15 bacterial genera. Heatmap shows the strength of association (Wilcoxon effect size) for the presence of each ARG with relative abundances of the 15 most abundant classified genera. *p*-values (FDR-corrected) are based on Wilcoxon rank-sum tests; ** indicates *p* < 0.01, * indicates *p* < 0.05. C) Proportion of infants in each treatment group in which antibiotic resistance genes were detected at given time points. Bar plots are colored according to treatment group (azithromycin or placebo). *P* values (FDR-corrected) are from Chi-squared tests; * indicates *p* < 0.05. D) Boxplots showing the total number of ARGs in treatment/probiotic subgroups (infants treated with azithromycin (with and without probiotics, AZM+Pro+and AZM+Pro-, respectively) or placebo (with and without probiotics, AZM- Pro+and AZM- Pro-, respectively)) at given time points, colored according to treatment/probiotic subgroup. Boxplots show median (center line) and interquartile ranges (IQR) (box limits); whiskers extend ± 1.5∗IQR from box's quartile. Points outside the whiskers represent outliers. E) Barplots showing the proportion (%) of infants in which *erm* (C) was detected, separated by treatment/probiotic subgroup and time point. *p* values (FDR-corrected) in D) are from Chi-square tests; * indicates *p* < 0.05, ** indicates *p* < 0.01. ARG, antibiotic resistance gene.

The presence of ARGs was weakly linked to specific taxa within the microbiome. Each ARG was significantly associated with at least one of the most abundant genera, with the greatest number of significant associations detected for *erm* (F) (Figure 5B). Effect sizes were generally small (ranging from < 0.01 to 0.26) for all associations except those involving *Enterococcus*. Increased relative abundance of *Enterococcus* was positively correlated with both *erm* (F) and *erm* (B) (effect sizes 0.33 and 0.30, respectively). Notably, when analyzes were conducted within treatment groups, the effect sizes for the associations of *Enterococcus* with both genes remained moderate in the azithromycin group, but were small in the placebo group (*p* values < 0.05).

### Impact of azithromycin treatment and probiotics on preterm macrolide resistance genes

After adjusting for DOL, azithromycin treatment was significantly positively associated with the total number of ARGs in comparison to infants given placebo (estimate = 0.364, SE = 0.165, z = 2.21, *p* = 0.028). By the days 14–21 time point, azithromycin-treated infants showed a trend towards increased prevalence of all macrolide-resistance genes, with *erm*(C) being significantly more prevalent in the azithromycin arm (*p* = 0.019; Figure 5C). The increase in *erm* (C) in azithromycin-treated infants was also observed after accounting for DOL in logistic regression models (estimate = 0.571, SE = 0.22, z = 2.596, *p* = 0.009).

Because of the impact of probiotic exposure on the microbiome, we next investigated whether probiotic exposure was related to the presence of ARGs. After adjusting for DOL, probiotic exposure was positively associated with the presence of *erm* (C), as well as with the total number of ARGs (*p* values < 0.05; Supplementary Table 6). The increased likelihood of detecting *erm* (C) and the total number of ARGs in probiotic-exposed infants remained significant in models even after adjusting for azithromycin treatment (*p* values < 0.01). Thus, we next explored both factors within the subgroups combining treatment status and probiotic exposure. There was no significant difference in the median number of ARGs (Kruskal‒Wallis *p*-values > 0.05), but the proportion of infants with *erm*(C) was highest in AZM+Pro+infants compared to all other groups at the day 10 time point, and compared to AZM- Pro- infants at days 14–21 (Chi-squared *p-*values < 0.05; Figure 5D and E).

### Association of preterm gut microbiome with clinical outcome data

Finally, we explored whether gut microbiome alterations mediated prevalent clinical outcomes in the cohort. Treatment for nosocomial infection, which typically involves antibiotic administration, was the only outcome significantly associated with bacterial alpha diversity after adjusting for co-variates, with treated infants having significantly lower Shannon diversity (*p* = 0.014; Supplementary Figure 6A). Treatment for patent ductus arteriosus (PDA) was associated with reduced relative abundances of Pseudomonadota phylum in infants (q value = 0.09; Supplementary Figure 6B). Infants who subsequently died before discharge had higher relative abundances of Pseudomonadota and *Enterobacter/Klebsiella*, and lower relative abundances of Bacillota and *Staphylococcus* in stool samples collected during the study period (q values < 0.25; Supplementary Figure 6C–F).

## Discussion

The current study sought to investigate the impact of an interventional randomized controlled trial of systemic azithromycin administration on the gut microbiomes and ARGs of 693 preterm infants from 28 neonatal intensive care units (NICUs). Azithromycin treatment led to reductions in alpha diversity and shifts in overall microbiota composition, characterized by decreased relative abundances of *Bifidobacterium* and increased abundances of pathobionts like *Staphylococcus* and *Enterobacter/Klebsiella*. Reduced bacterial richness and/or diversity during infancy have been previously linked to adverse health outcomes, including life-threatening illnesses such as necrotizing enterocolitis (NEC)^43^ ^,^ ^44^ and late-onset sepsis (LOS),^45^ as well as increased risks of later-life type 1 diabetes^46^ and allergic diseases, including asthma, eczema, allergic rhinitis, and food allergies.^47-51^ Although we did not find any significant associations between alpha diversity and selected health outcomes at 36 weeks' postmenstrual age in this study, we cannot exclude the possibility of long-term impacts.

Given that azithromycin is a broad-spectrum antibiotic, it was not surprising to observe effects beyond its intended activity against pulmonary *Ureaplasma*. Most notably, the reduction in the relative abundance and prevalence of *Bifidobacterium* in response to azithromycin, which was further corroborated by *in vitro* microbiological sensitivity assays. This is in accordance with previous studies in preterm and full-term infants that have also shown reduced abundances of *Bifidobacterium* following antibiotic exposure,^6^ ^,^ ^12^ ^,^ ^15^ ^,^ ^18^ ^,^ ^52^ including the use of macrolides.^53^ *Bifidobacterium* spp. are considered beneficial to infant health,^5^ largely responsible for the breakdown of human milk oligosaccharides (HMOs)^54^ and production of short-chain fatty acids.^55^ ^,^ ^56^ Given their important roles in training the immune system and gut barrier function,^57^ ^,^ ^58^ depletion of *Bifidobacterium* spp. during infancy may lead to increased risks of NEC and later life inflammatory and metabolic diseases.^58-60^ Similarly, the prevalence of *Clostridium sensu stricto*, also reported to metabolize HMOs and be potentially health-associated,^61^ increased over time in placebo-treated infants, but not in azithromycin-treated infants. While we lack species- and strain-level resolution, we did find differential strain sensitivities *in vitro*, supporting previous studies showing that *Bifidobacterium* species exhibit varying susceptibilities to macrolide treatments,^62^ ^,^ ^63^ including azithromycin.^64^

The azithromycin-induced reduction in commensal *Bifidobacterium* may have promoted the expansion of pathobionts by compromising key defensive mechanisms, including competitive exclusion, production of antimicrobial substances like acids and bacteriocins, and immune modulation,^65^ or via unknown interspecies interaction mechanisms.^66^ This is particularly concerning in vulnerable preterm infants, who are frequently colonized by environmental microbes and exposed to opportunistic pathogens in NICUs.^67^ The *Staphylococcus* and *Enterobacter/Klebsiella* genera contain members of the ESKAPE group, a family of pathogens frequently implicated in nosocomial infections and known for their multidrug resistance and subsequent severe health outcomes.^68^ Indeed, our analysis identified significant positive associations between the relative abundances of both *Staphylococcus* and *Enterobacter/Klebsiella* and pre-discharge mortality. Higher *Klebsiella* has also been associated with development of NEC.^69^

In addition to its impact on the preterm gut microbiome, azithromycin treatment increased the total number of macrolide-resistant genes, particularly *erm*(C). Our findings align with a systematic review reporting azithromycin treatment consistently increased macrolide resistance gene abundance in children under 2 y.^16^ Our study extends these findings by confirming this effect specifically in preterm infants, a population already at heightened risk of antibiotic resistance due to both their immaturity and complex clinical management necessitating relatively high antibiotic use. *erm* (C) was not linked to a specific genus, suggesting that *erm* (C) was broadly distributed across multiple genera or partially carried on mobile genetic elements.^70^

The effects of azithromycin were still evident at DOL 14–21, following cessation of the treatment on DOL 10. As we did not observe recovery of the gut microbiome or ARGs during the study period, the long-term persistence of azithromycin's effects remains uncertain. Previous research suggests that antibiotic exposure can lead to persistent alterations lasting for at least several months.^8^ ^,^ ^53^ ^,^ ^71-73^ Given the prolonged azithromycin exposure in our study (10 d), the potential for sustained effects may be even greater, as supported by previous studies showing prolonged reduced microbial diversity and *Bifidobacterium* in infants exposed to antibiotics for ≥5 d.^10^ ^,^ ^15^

Overall patterns were similar to those observed in previous studies of preterm infants, especially that site and probiotic use were key factors shaping the gut microbiome.^17^ ^,^ ^18^ Notably, site and probiotic variables are not independent, since most units who use probiotics offer them to “all” preterm infants, and those who do not use generally never use them. There is also a propensity for cross-colonization of probiotics within NICUs.^74^ In accordance with our findings, other studies have shown probiotics administered to preterm infants increase the relative abundance of *Bifidobacterium* spp., including those not contained in the product, as well as reduce the abundance of pathobionts.^18^ ^,^ ^75-78^ We also confirmed previous findings that probiotics do not fully restore the relative abundance of *Bifidobacterium* or completely reverse microbial alterations when administered to infants during or after antibiotic courses,^75^ ^,^ ^76^ ^,^ ^79^ ^,^ ^80^ likely due to antibiotic-mediated disruption of probiotic colonization.

We found probiotics in combination with azithromycin may potentiate the expansion of *Enterobacter/Klebsiella* and increase the frequency of *erm*(C) resistance gene and the total number of macrolide-resistant genes, beyond what is observed with azithromycin alone. Although we did not see a direct association between *Bifidobacterium* and *erm* (C), there is evidence that some probiotic products contain ARGs,^81^ ^,^ ^82^though the specific presence of *erm* (C) has not been reported.^63^ Similar to our study, Yuu et al. ^80^found that preterm infants receiving both antibiotics and probiotics had the highest percentages of ARGs compared to either treatment alone.^80^ This contrasts with several studies reporting reduced abundance and/or diversity of ARGs in preterm infants receiving probiotics.^77^ ^,^ ^83-86^ These conflicting findings between studies may reflect the lack of power in the cohorts when stratifying into groups and highlight the need for further research into these interactions, their impact on microbial community dynamics, and their potential role in driving AMR.

To the best of our knowledge, the current study represents the largest multi-site (*n* = 28 NICUs) study of gut microbiome in preterm infants and the most extensive analysis of infants in the first three weeks. Notwithstanding, there are several limitations. Because this study focused on early time windows, the longer-term impacts on microbiome development could not be explored. Related, owing to the low microbial biomass following birth, despite applying a low rarefaction threshold of 1000 reads, most baseline samples had insufficient sequencing depth to be included in the analysis. Furthermore, some variables such as feeding information, medication, and caffeine were not routinely collected; however, they are expected to be comparable across sites (i.e., all units used donor human milk and caffeine use is routine in the UK). Probiotics were associated with the gut microbiome, but most probiotic infants (84%) received Labinic, precluding analysis stratified by formulation to determine potential strain-specific microbiome effects. Furthermore, the V3–V4 16S rRNA gene sequencing approach may have limited sensitivity for low-abundance *Lactobacillus* taxa, potentially explaining challenges in identifying these taxa. Relatedly, given the lack of natural Bifidobacterium in the sampling period, we are unable to disentangle whether azithromycin directly suppresses this genus or interferes with probiotic-driven engraftment. Further, probiotic administration was not randomized, meaning these findings should be regarded as observational. Lastly, for analyzes relating microbiome features to clinical outcomes, microbiome measurements were derived from stool samples collected during the first 21 d of life. Because some clinical outcomes could occur during or after this sampling period, these analyzes should be interpreted as exploratory associations and not as evidence of a temporal or causal relationship.

In summary, this study provides valuable insights that could inform future research and guide clinical decision-making. Given the detrimental effects of azithromycin on both microbial communities and ARGs, our findings suggest that this antibiotic may not be an optimal choice for preterm infants. The results emphasize the need for caution when using antibiotics in vulnerable populations and prompt further consideration of the impact of combining probiotics and antibiotics, as their joint effects on microbial community dynamics and ARGs remain unclear. It also emphasize the importance of exploring alternative antimicrobial strategies, such as bacteriocins or postbiotics. The site-specific impacts on gut microbiome development further emphasize the potential challenges in generalizing findings across NICUs. Further research is needed to better understand the ecological impacts of microbiome-altering drugs and their long-term consequences in vulnerable populations.

## Supplementary Material

Supplementary Figure 5.pdf Supplementary Figure 5.pdf

Supplementary Figure1.pdf Supplementary Figure 1.pdf

Supplementary Figure 3.pdf Supplementary Figure 3.pdf

Supplementary Figure 6.pdf Supplementary Figure 6.pdf

Supplementary Table 6.csv Supplementary Table 6.csv

Supplementary Table 5.csv Supplementary Table 5.csv

Supplementary Table 1.csv Supplementary Table 1.csv

Supplementary Table 4.csv Supplementary Table 4.csv

Supplementary Table 2.csv Supplementary Table 2.csv

Supplementary Figure 2.pdf Supplementary Figure 2.pdf

Supplementary Table 3.csv Supplementary Table 3.csv

Supplementary Figure 4.pdf

Supplementary Figure Caption Supplementary Figure Caption

## Data access

All sequencing data generated and analyzed in the present study have been deposited in the European Nucleotide Archive under study accession number PRJEB111179.

## References

[1] Bonadies L , Zaramella P , Porzionato A , Perilongo G , Muraca M , Baraldi E . Present and future of bronchopulmonary dysplasia. J Clin Med. 2020;9(5):1539. doi: 10.3390/jcm9051539. PMC7290764 32443685

[2] Lowe J , Watkins WJ , Edwards MO , Spiller OB , Jacqz-Aigrain E , Kotecha SJ , Kotecha S . Association between pulmonary ureaplasma colonization and bronchopulmonary dysplasia in preterm infants. Pediatr Infect Dis J. 2014;33(7):697–702. doi: 10.1097/INF.0000000000000239. 24445836

[3] Lowe J , Gillespie D , Hubbard M , Zhang L , Kirby N , Pickles T , Thomas-Jones E , Turner MA , Klein N , Marchesi JR , et al. Study protocol: azithromycin therapy for chronic lung disease of prematurity (AZTEC) - a randomised, placebo-controlled trial of azithromycin for the prevention of chronic lung disease of prematurity in preterm infants. BMJ Open. 2020;10(10):e041528. doi: 10.1136/bmjopen-2020-041528. PMC7539578 33028566

[4] Lowe J , Gillespie D , Aboklaish A , Lau TMM , Consoli C , Babu M , Goddard M , Hood K , Klein N , Thomas-Jones E . Azithromycin therapy for prevention of chronic lung disease of prematurity (AZTEC): a multicentre, double-blind, randomised, placebo-controlled trial. The Lancet Respiratory Medicine. 2024;12:608–618. doi: 10.1016/S2213-2600(24)00079-1. 38679042

[5] Sarkar A , Yoo JY , Valeria Ozorio Dutra S , Morgan KH , Groer M . The association between early-life gut microbiota and long-term health and diseases. J Clin Med. 2021;10(3):459. doi: 10.3390/jcm10030459. PMC7865818 33504109

[6] Chang H-Y , Ho Y-H , Chang J-H , Tsai K-N , Liu C-Y , Hsu C-H , Lin C-Y , Ko MH-J , Lee H-C . Impact of early empiric antibiotic regimens on the gut microbiota in very low birth weight preterm infants: an observational study. Front Pediatr. 2021;9:651713. doi: 10.3389/fped.2021.651713. PMC8200535 34136438

[7] Dardas M , Gill SR , Grier A , Pryhuber GS , Gill AL , Lee Y-H , Guillet R . The impact of postnatal antibiotics on the preterm intestinal microbiome. Pediatr Res. 2014;76:150–158. doi: 10.1038/pr.2014.69. 24819377

[8] Gasparrini AJ , Wang B , Sun X , Kennedy EA , Hernandez-Leyva A , Ndao IM , Tarr PI , Warner BB , Dantas G . Persistent metagenomic signatures of early-life hospitalization and antibiotic treatment in the infant gut microbiota and resistome. Nat Microbiol. 2019;4:2285–2297. doi: 10.1038/s41564-019-0550-2. PMC6879825 31501537

[9] Gibson MK , Wang B , Ahmadi S , Burnham C-AD , Tarr PI , Warner BB , Dantas G . Developmental dynamics of the preterm infant gut microbiota and antibiotic resistome. Nat Microbiol. 2016;1:16024. doi: 10.1038/nmicrobiol.2016.24. PMC5031140 27572443

[10] Greenwood C , Morrow AL , Lagomarcino AJ , Altaye M , Taft DH , Yu Z , Newburg DS , Ward DV , Schibler KR . Early empiric antibiotic use in preterm infants is associated with lower bacterial diversity and higher relative abundance of enterobacter. J Pediatr. 2014;165(1):23–29. doi: 10.1016/j.jpeds.2014.01.010. PMC4074569 24529620

[11] Jia J , Xun P , Wang X , He K , Tang Q , Zhang T , Wang Y , Tang W , Lu L , Yan W , et al. Impact of postnatal antibiotics and parenteral nutrition on the gut microbiota in preterm infants during early life. J Parenter Enter Nutr. 2020;44(4):639–654. doi: 10.1002/jpen.1695. 31452218

[12] Reyman M , van Houten MA , Watson RL , Chu MLJN , Arp K , de Waal WJ , Schiering I , Plötz FB , Willems RJL , van Schaik W , et al. Effects of early-life antibiotics on the developing infant gut microbiome and resistome: a randomized trial. Nat Commun. 2022;13:893. doi: 10.1038/s41467-022-28525-z. PMC8850541 35173154

[13] Rooney AM , Timberlake K , Brown KA , Bansal S , Tomlinson C , Lee K-S , Science M , Coburn B . Each additional day of antibiotics is associated with lower gut anaerobes in neonatal intensive care unit patients. Clin Infect Dis. 2020;70(12):2553–2560. doi: 10.1093/cid/ciz69. PMC7286368 31367771

[14] Zhu D , Xiao S , Yu J , Ai Q , He Y , Cheng C , Zhang Y , Pan Y . Effects of one-week empirical antibiotic therapy on the early development of gut microbiota and metabolites in preterm infants. Sci Rep. 2017;7:8025. doi: 10.1038/s41598-017-08530-9. PMC5556106 28808302

[15] Zwittink RD , Renes IB , van Lingen RA , van Zoeren-Grobben D , Konstanti P , Norbruis OF , Martin R , Groot Jebbink LJM , Knol J , Belzer C . Association between duration of intravenous antibiotic administration and early-life microbiota development in late-preterm infants. Eur J Clin Microbiol Infect Dis. 2018;37:475–483. doi: 10.1007/s10096-018-3193-y. PMC5816780 29368074

[16] Luchen CC , Chibuye M , Spijker R , Simuyandi M , Chisenga C , Bosomprah S , Chilengi R , Schultsz C , Mende DR , Harris VC . Impact of antibiotics on gut microbiome composition and resistome in the first years of life in low- to middle-income countries: a systematic review. PLoS Med. 2023;20(6):e1004235. doi: 10.1371/journal.pmed.1004235. PMC10298773 37368871

[17] Embleton N , Berrington J , Cummings S , Dorling J , Ewer A , Frau A , Juszczak E , Kirby J , Lamb C , Lanyon C , et al. Lactoferrin impact on gut microbiota in preterm infants with late-onset sepsis or necrotising enterocolitis: the MAGPIE mechanisms of action study. Efficacy and Mechanism Evaluation. 2021;8(14):1–88. doi: 10.3310/eme08140. 34591437

[18] Beck LC , Masi AC , Young GR , Vatanen T , Lamb CA , Smith R , Coxhead J , Butler A , Marsland BJ , Embleton ND , et al. Strain-specific impacts of probiotics are a significant driver of gut microbiome development in very preterm infants. Nat Microbiol. 2022;7:1525–1535. doi: 10.1038/s41564-022-01213-w. PMC9519454 36163498

[19] Comeau AM , Douglas GM , Langille MGI . Microbiome helper: a custom and streamlined workflow for microbiome research. mSystems. 2017;2(1), e00127. 10.1128/msystems.00127-16. PMC5209531 28066818

[20] Comeau AM , Kwawukume A . Preparing multiplexed 16S/18S/ITS amplicons for the illumina MiSeq. protocols.io. 2023. doi: 10.17504/protocols.io.4r3l277k3g1y/v1 [Accessed 05 June 2025].

[21] Bolyen E , Rideout JR , Dillon MR , Bokulich NA , Abnet CC , Al-Ghalith GA , Alexander H , Alm EJ , Arumugam M , Asnicar F , et al. Reproducible, interactive, scalable and extensible microbiome data science using QIIME 2. NatBi. 2019;37:852–857. doi: 10.1038/s41587-019-0209-9. PMC7015180 31341288

[22] Callahan BJ , McMurdie PJ , Rosen MJ , Han AW , Johnson AJA , Holmes SP . DADA2: high-resolution sample inference from illumina amplicon data. Nat Methods. 2016;13:581–583. doi: 10.1038/nmeth.3869. PMC4927377 27214047

[23] Rognes T , Flouri T , Nichols B , Quince C , Mahé F . VSEARCH: a versatile open source tool for metagenomics. PeerJ. 2016;4:e2584. doi: 10.7717/peerj.2584. PMC5075697 27781170

[24] Quast C , Pruesse E , Yilmaz P , Gerken J , Schweer T , Yarza P , Peplies J , Glöckner FO . The SILVA ribosomal RNA gene database project: improved data processing and web-based tools. Nucleic Acids Res. 2013;41(D1):D590–D596. doi: 10.1093/nar/gks1219. PMC3531112 23193283

[25] Oksanen J , Blanchet FG , Friendly M , Kindt R , Legendre P , McGlinn D , Minchin PR , O’Hara RB , Simpson GL , Solymos P , et al. 2020. vegan: Community Ecology Package (version 2.6.4) [R package]. Available at: https://CRAN.R-project.org/package=vegan [Accessed 21 May 2025]

[26] Gallacher DJ , Zhang L , Aboklaish AF , Mitchell E , Wach R , Marchesi JR , Kotecha S . Baseline azithromycin resistance in the gut microbiota of preterm born infants. Pediatr Res. 2024;95:205–212. doi: 10.1038/s41390-023-02743-7. PMC10798878 37550487

[27] Sanchez ML , Jones RN . E test, an antimicrobial susceptibility testing method with broad clinical and epidemiologic application. Antimicrobic Newsletter. 1992;8(1):1–7. doi: 10.1016/0738-1751(92)90015-3.

[28] EUCAST . 2025a. The European Committee on Antimicrobial Susceptibility Testing. Routine and extended internal quality control for MIC determination and disk diffusion as recommended by EUCAST. Version 15.0, 2025[online] Available at: https://www.eucast.org [Accessed 21 May 2025]

[29] EUCAST . 2025b. The European Committee on Antimicrobial Susceptibility Testing. Breakpoint tables for interpretation of MICs and zone diameters. Version 15.0, 2025. [online]. Available at: https://www.eucast.org [Accessed 21 May 2025].

[30] R Core Team R: A language and environment for statistical computing. 2021. Vienna, Austria: R Foundation for Statistical Computing https://www.R-project.org/ [Accessed 21 May 2025].

[31] Wickham H . 2016. ggplot2: Elegant Graphics for Data Analysis (version 3.4.3) [R package]. doi: 10.1007/978-3-319-24277-4 [Accessed 21 May 2025]

[32] Mallick H , Rahnavard A , McIver LJ , Ma S , Zhang Y , Nguyen LH , Tickle TL , Weingart G , Ren B , Schwager EH , et al. Multivariable association discovery in population-scale meta-omics studies. PLoS Comput Biol. 2021;17(11):e1009442. doi: 10.1371/journal.pcbi.1009442. PMC8714082 34784344

[33] Benjamini Y , Hochberg Y . Controlling the false discovery rate: a practical and powerful approach to multiple testing. J R Stat Soc, B: Stat Methodol. 1995;57(1):289–300. doi: 10.1111/j.2517-6161.1995.tb02031.x.

[34] Kassambara A . 2023. rstatix: Pipe-Friendly Framework for Basic Statistical Tests (version 0.7.2) [R package]. Available at: https://CRAN.R-project.org/package=rstatix [Accessed 21 May2025]

[35] Bates D , Maechler M , Bolker B , Walker S . lme4: Linear Mixed-Effects Models using ‘Eigen’ and S4 (version 1.1.35.5) [R package]. 2024 Available at: https://cran.r-project.org/web/packages/lme4/ [Accessed 21 May 2025].

[36] Ripley B , Venables B , Bates DM , Hornik K , Gebhardt A , Firth D . 2024. MASS: Support Functions and Datasets for Venables and Ripley’s MASS (version 7.3.60.0.1) [R package]. Available at: https://CRAN.R-project.org/package=MASS [Accessed 21 May 2025]

[37] Fox J , Weisberg S . An R Companion to Applied Regression. 2019. R package car version 3.1.2 Available at: https://CRAN.R-project.org/package=car [Accessed 21 May 2025].

[38] Lenth RV . 2024. emmeans: Estimated Marginal Means, aka Least-Squares Means (version 1.10.0) [R package]. Available at: https://CRAN.R-project.org/package=emmeans . [Accessed 21 May 2025]

[39] Hamidi B , Wallace K , Vasu C , Alekseyenko AV . $W_{d}^{*}$-test: robust distance-based multivariate analysis of variance. Microbiome. 2019;7:51. doi: 10.1186/s40168-019-0659-9. PMC6444669 30935409

[40] Christensen R . ordinal—Regression Models for Ordinal Data (version 2023.12.4) [R package]. 2023 Available at https://CRAN.R-project.org/package=ordinal [Accessed 12 May 2025].

[41] Spangler SK , Jacobs MR , Appelbaum PC . Susceptibilities of 201 anaerobes to erythromycin, azithromycin, clarithromycin, and roxithromycin by oxyrase agar dilution and E test methodologies. J Clin Microbiol. 1995;33(5):1366–1367. doi: 10.1128/jcm.33.5.1366-1367.1995. PMC228167 7615756

[42] Wood DE , Lu J , Langmead B . Improved metagenomic analysis with kraken 2. Genome Biol. 2019;20:257. doi: 10.1186/s13059-019-1891-0. PMC6883579 31779668

[43] Thänert R , Schwartz DJ , Keen EC , Hall-Moore C , Wang B , Shaikh N , Ning J , Rouggly-Nickless LC , Thänert A , Ferreiro A , et al. Clinical sequelae of gut microbiome development and disruption in hospitalized preterm infants. Cell Host Microbe. 2024;32(10):1822–1837.e5. doi: 10.1016/j.chom.2024.07.027. PMC11466706 39197454

[44] Warner BB , Deych E , Zhou Y , Hall-Moore C , Weinstock GM , Sodergren E , Shaikh N , Hoffmann JA , Linneman LA , Hamvas A , et al. Gut bacteria dysbiosis and necrotising enterocolitis in very low birthweight infants: a prospective case-control study. Lancet. 2016;387(10031):1928–1936. doi: 10.1016/S0140-6736(16)00081-7. PMC5553277 26969089

[45] Graspeuntner S , Waschina S , Künzel S , Twisselmann N , Rausch TK , Cloppenborg-Schmidt K , Zimmermann J , Viemann D , Herting E , Göpel W , et al. Gut dysbiosis with bacilli dominance and accumulation of fermentation products precedes late-onset sepsis in preterm infants. Clin Infect Dis. 2019;69(2):268–277. doi: 10.1093/cid/ciy882. 30329017

[46] Kostic AD , Gevers D , Siljander H , Vatanen T , Hyötyläinen T , Hämäläinen A-M , Peet A , Tillmann V , Pöhö P , Mattila I , et al. The dynamics of the human infant gut microbiome in development and in progression toward type 1 diabetes. Cell Host Microbe. 2015;17(2):260–273. doi: 10.1016/j.chom.2015.01.001. PMC4689191 25662751

[47] Abrahamsson TR , Jakobsson HE , Andersson AF , Björkstén B , Engstrand L , Jenmalm MC . Low diversity of the gut microbiota in infants with atopic eczema. J Allergy Clin Immunol. 2012;129(2):434–440.e2. doi: 10.1016/j.jaci.2011.10.025. 22153774

[48] Abrahamsson TR , Jakobsson HE , Andersson AF , Björkstén B , Engstrand L , Jenmalm MC . Low gut microbiota diversity in early infancy precedes asthma at school age. Clin Exp Allergy. 2014;44(6):842–850. doi: 10.1111/cea.12253. 24330256

[49] Azad MB , Konya T , Guttman DS , Field CJ , Sears MR , HayGlass KT , Mandhane PJ , Turvey SE , Subbarao P , Becker AB , et al. Infant gut microbiota and food sensitization: associations in the first year of life. Clin Exp Allergy. 2015;45(3):632–643. doi: 10.1111/cea.12487. 25599982

[50] Hoskinson C , Dai DLY , Del Bel KL , Becker AB , Moraes TJ , Mandhane PJ , Finlay BB , Simons E , Kozyrskyj AL , Azad MB , et al. Delayed gut microbiota maturation in the first year of life is a hallmark of pediatric allergic disease. Nat Commun. 2023;14:4785. doi: 10.1038/s41467-023-40336-4. PMC10465508 37644001

[51] Ismail IH , Oppedisano F , Joseph SJ , Boyle RJ , Licciardi PV , Robins‐ Browne RM , Tang MLK . Reduced gut microbial diversity in early life is associated with later development of eczema but not atopy in high‐ risk infants. Pediatr Allergy Immunol. 2012;23(7):674–681. doi: 10.1111/j.1399-3038.2012.01328.x. 22831283

[52] Chernikova DA , Madan JC , Housman ML , Zain-ul-abideen M , Lundgren SN , Morrison HG , Sogin ML , Williams SM , Moore JH , Karagas MR , et al. The premature infant gut microbiome during the first 6 weeks of life differs based on gestational maturity at birth. Pediatr Res. 2018;84:71–79. doi: 10.1038/s41390-018-0022-z. PMC6082716 29795209

[53] Korpela K , Salonen A , Saxen H , Nikkonen A , Peltola V , Jaakkola T , de Vos W , Kolho K-L . Antibiotics in early life associate with specific gut microbiota signatures in a prospective longitudinal infant cohort. Pediatr Res. 2020;88:438–443. doi: 10.1038/s41390-020-0761-5. 31954376

[54] Masi AC , Stewart CJ . Untangling human milk oligosaccharides and infant gut microbiome. iSci. 2022;25(1):103542. doi: 10.1016/j.isci.2021.103542. PMC8671521 34950861

[55] Koh A , De Vadder F , Kovatcheva-Datchary P , Bäckhed F . From dietary fiber to host physiology: short-chain fatty acids as key bacterial metabolites. Cell. 2016;165(6):1332–1345. doi: 10.1016/j.cell.2016.05.041. 27259147

[56] Tsukuda N , Yahagi K , Hara T , Watanabe Y , Matsumoto H , Mori H , Higashi K , Tsuji H , Matsumoto S , Kurokawa K , et al. Key bacterial taxa and metabolic pathways affecting gut short-chain fatty acid profiles in early life. ISME J. 2021;15(9):2574–2590. doi: 10.1038/s41396-021-00937-7. PMC8397723 33723382

[57] Ewaschuk JB , Diaz H , Meddings L , Diederichs B , Dmytrash A , Backer J , Looijer-van Langen M , Madsen KL . Secreted bioactive factors from bifidobacterium infantis enhance epithelial cell barrier function. Am J Physiol Gastrointest Liver Physiol. 2008;295(5):G1025–G1034. doi: 10.1152/ajpgi.90227.2008. 18787064

[58] Henrick BM , Rodriguez L , Lakshmikanth T , Pou C , Henckel E , Arzoomand A , Olin A , Wang J , Mikes J , Tan Z , et al. Bifidobacteria-mediated immune system imprinting early in life. Cell. 2021;184(15):3884–3898.e11. doi: 10.1016/j.cell.2021.05.030. 34143954

[59] Korpela K , Zijlmans MAC , Kuitunen M , Kukkonen K , Savilahti E , Salonen A , de Weerth C , de Vos WM . Childhood BMI in relation to microbiota in infancy and lifetime antibiotic use. Microbiome. 2017;5:26. doi: 10.1186/s40168-017-0245-y. PMC5335838 28253911

[60] Stanislawski MA , Dabelea D , Wagner BD , Iszatt N , Dahl C , Sontag MK , Knight R , Lozupone CA , Eggesbø M . Gut microbiota in the first 2 years of life and the association with body mass index at age 12 in a Norwegian birth cohort. mBio. 2018;9(5):e01751-18. doi: 10.1128/mBio.01751-18. PMC6199494 30352933

[61] Chapman JA , Masi AC , Beck LC , Watson H , Young GR , Browne HP , Shao Y , Kiu R , Nelson A , Doyle JA , et al. Clostridia from preterm infants metabolize human milk oligosaccharides to suppress pathobionts and modulate intestinal function in organoids. Nat Microbiol. 2026;11(4):940–959. doi: 10.1038/s41564-026-02297-4. PMC13056567 41840217

[62] Kammara R , Nellikka A . Acquiring bifidobacteria species from formula-fed and breast-fed newborns: identifying, quantifying and creating an antibiogram. Access Microbiology. 2023;5(8):acmi000590.v3. doi: 10.1099/acmi.0.000590.v3. PMC10484311 37691835

[63] Pei Z , Liu Y , Zhao F , Wang H , Zhao J , Chen W , Lu W . Antibiotic susceptibility testing and establishment of tentative species-specific microbiological cut-off values for bifidobacteria isolated from Chinese population. Probiotics Antimicrob Proteins. 2024;16:1657–1672. doi: 10.1007/s12602-023-10128-9. 37515711

[64] Neut C , Mahieux S , Dubreuil LJ . Antibiotic susceptibility of probiotic strains: is it reasonable to combine probiotics with antibiotics?. Med Mal Infect. 2017;47(7):477–483. doi: 10.1016/j.medmal.2017.07.001. 28797834

[65] Pickard JM , Zeng MY , Caruso R , Núñez G . Gut microbiota: role in pathogen colonization, immune responses, and inflammatory disease. Immunol Rev. 2017;279(1):70–89. doi: 10.1111/imr.12567. PMC5657496 28856738

[66] Bottery MJ , Matthews JL , Wood AJ , Johansen HK , Pitchford JW , Friman V-P . Inter-species interactions alter antibiotic efficacy in bacterial communities. ISME J. 2022;16:812–821. doi: 10.1038/s41396-021-01130-6. PMC8857223 34628478

[67] Brooks B , Firek BA , Miller CS , Sharon I , Thomas BC , Baker R , Morowitz MJ , Banfield JF . Microbes in the neonatal intensive care unit resemble those found in the gut of premature infants. Microbiome. 2014;2(1):1. doi: 10.1186/2049-2618-2-1. PMC4392516 24468033

[68] Miller WR , Arias CA . ESKAPE pathogens: antimicrobial resistance, epidemiology, clinical impact and therapeutics. Nat Rev Microbiol. 2024;22:598–616. doi: 10.1038/s41579-024-01054-w. PMC13147291 38831030

[69] Olm MR , Bhattacharya N , Crits-Christoph A , Firek BA , Baker R , Song YS , Morowitz MJ , Banfield JF . Necrotizing enterocolitis is preceded by increased gut bacterial replication, Klebsiella, and fimbriae-encoding bacteria. Sci Adv. 2019;5(12):eaax5727–eaax5727. doi: 10.1126/sciadv.aax5727. PMC6905865 31844663

[70] Partridge SR , Kwong SM , Firth N , Jensen SO . Mobile genetic elements associated with antimicrobial resistance. Clin Microbiol Rev. 2018;31(4):e00088-17. doi: 10.1128/CMR.00088-17. PMC6148190 30068738

[71] Hutchinson RA , Costeloe KL , Wade WG , Millar MR , Ansbro K , Stacey F , Fleming PF . Intravenous antibiotics in preterm infants have a negative effect upon microbiome development throughout preterm life. Gut Pathog. 2023;15:18. doi: 10.1186/s13099-023-00544-1. PMC10120188 37085896

[72] Li X , Stokholm J , Brejnrod A , Vestergaard GA , Russel J , Trivedi U , Thorsen J , Gupta S , Hjelmsø MH , Shah SA , et al. The infant gut resistome associates with E. Coli, environmental exposures, gut microbiome maturity, and asthma-associated bacterial composition. Cell Host Microbe. 2021;29(6):975–987.e4. doi: 10.1016/j.chom.2021.03.017. 33887206

[73] Uzan-Yulzari A , Turta O , Belogolovski A , Ziv O , Kunz C , Perschbacher S , Neuman H , Pasolli E , Oz A , Ben-Amram H , et al. Neonatal antibiotic exposure impairs child growth during the first six years of life by perturbing intestinal microbial colonization. Nat Commun. 2021;12:443. doi: 10.1038/s41467-020-20495-4. PMC7838415 33500411

[74] Costeloe K , Bowler U , Brocklehurst P , Hardy P , Heal P , Juszczak E , King A , Panton N , Stacey F , Whiley A , et al. A randomised controlled trial of the probiotic bifidobacterium breve BBG-001 in preterm babies to prevent sepsis, necrotising enterocolitis and death: the probiotics in preterm infantS (PiPS) trial. Health Technol Assess. 2016;20(66):1–194. doi: 10.3310/hta20660. PMC5027379 27594381

[75] Alcon-Giner C , Dalby MJ , Caim S , Ketskemety J , Shaw A , Sim K , Lawson MAE , Kiu R , Leclaire C , Chalklen L , et al. Microbiota supplementation with bifidobacterium and lactobacillus modifies the preterm infant gut microbiota and metabolome: an observational study. Cell Reports Medicine. 2020;1(5):100077. doi: 10.1016/j.xcrm.2020.100077. PMC7453906 32904427

[76] Esaiassen E , Hjerde E , Cavanagh JP , Pedersen T , Andresen JH , Rettedal SI , Støen R , Nakstad B , Willassen NP , Klingenberg C . Effects of probiotic supplementation on the gut microbiota and antibiotic resistome development in preterm infants. Front Pediatr. 2018;6:347. doi: 10.3389/fped.2018.00347. PMC6250747 30505830

[77] Nguyen M , Holdbrooks H , Mishra P , Abrantes MA , Eskew S , Garma M , Oca C-G , McGuckin C , Hein CB , Mitchell RD , et al. Impact of probiotic B. Infantis EVC001 feeding in premature infants on the gut microbiome, nosocomially acquired antibiotic resistance, and enteric inflammation. Front Pediatr. 2021;9:618009. doi: 10.3389/fped.2021.618009. PMC7921802 33665175

[78] van Best N , Trepels-Kottek S , Savelkoul P , Orlikowsky T , Hornef MW , Penders J . Influence of probiotic supplementation on the developing microbiota in human preterm neonates. Gut Microbes. 2020;12(1):1826747. doi: 10.1080/19490976.2020.1826747. PMC7588225 33095113

[79] Tapiainen T , Koivusaari P , Brinkac L , Lorenzi HA , Salo J , Renko M , Pruikkonen H , Pokka T , Li W , Nelson K , et al. Impact of intrapartum and postnatal antibiotics on the gut microbiome and emergence of antimicrobial resistance in infants. Sci Rep. 2019;9:10635. doi: 10.1038/s41598-019-46964-5. PMC6650395 31337807

[80] Yuu EY , Bührer C , Eckmanns T , Fulde M , Herz M , Kurzai O , Lindstedt C , Panagiotou G , Piro VC , Radonic A , et al. The gut microbiome, resistome, and mycobiome in preterm newborn infants and mouse pups: lack of lasting effects by antimicrobial therapy or probiotic prophylaxis. Gut Pathog. 2024;16:27. doi: 10.1186/s13099-024-00616-w. PMC11089716 38735967

[81] Gueimonde M , Sánchez B , G. de los Reyes-Gavilán C , Margolles A . Antibiotic resistance in probiotic bacteria. Front Microbiol. 2013;4:202. doi: 10.3389/fmicb.2013.00202. PMC3714544 23882264

[82] Montassier E , Valdés-Mas R , Batard E , Zmora N , Dori-Bachash M , Suez J , Elinav E . Probiotics impact the antibiotic resistance gene reservoir along the human GI tract in a person-specific and antibiotic-dependent manner. Nat Microbiol. 2021;6:1043–1054. doi: 10.1038/s41564-021-00920-0. PMC8318886 34226711

[83] Bargheet A , Klingenberg C , Esaiassen E , Hjerde E , Cavanagh JP , Bengtsson-Palme J , Pettersen VK . Development of early life gut resistome and mobilome across gestational ages and microbiota-modifying treatments. EBioMedicine. 2023;92:104613. doi: 10.1016/j.ebiom.2023.104613. PMC10192547 37187112

[84] Casaburi G , Duar RM , Vance DP , Mitchell R , Contreras L , Frese SA , Smilowitz JT , Underwood MA . Early-life gut microbiome modulation reduces the abundance of antibiotic-resistant bacteria. Antimicrobial Resistance & Infection Control. 2019;8:131. doi: 10.1186/s13756-019-0583-6. PMC6693174 31423298

[85] Guitor AK , Yousuf EI , Raphenya AR , Hutton EK , Morrison KM , McArthur AG , Wright GD , Stearns JC . Capturing the antibiotic resistome of preterm infants reveals new benefits of probiotic supplementation. Microbiome. 2022;10:136. doi: 10.1186/s40168-022-01327-7. PMC9414150 36008821

[86] Kiu R , Darby EM , Alcon-Giner C , Acuna-Gonzalez A , Camargo A , Lamberte LE , Phillips S , Sim K , Shaw AG , Clarke P , et al. Impact of early life antibiotic and probiotic treatment on gut microbiome and resistome of very-low-birth-weight preterm infants. Nat Communications. 2025;14(16(1)):7569. doi: 10.1101/2024.12.18.629205. PMC12354744 40813371

