# Supplementary material for: The impact of prophylactic azithromycin on the developing preterm gut microbiome and resistome
Source: Gut Microbes. 2026 Oct 15;18(1):2748147. doi: 10.1080/19490976.2026.2748147 (PMC13679317; doi:10.1080/19490976.2026.2748147)
Supplement: Supplementary file 1 [file KGMI_A_2748147_SM8532.pdf]

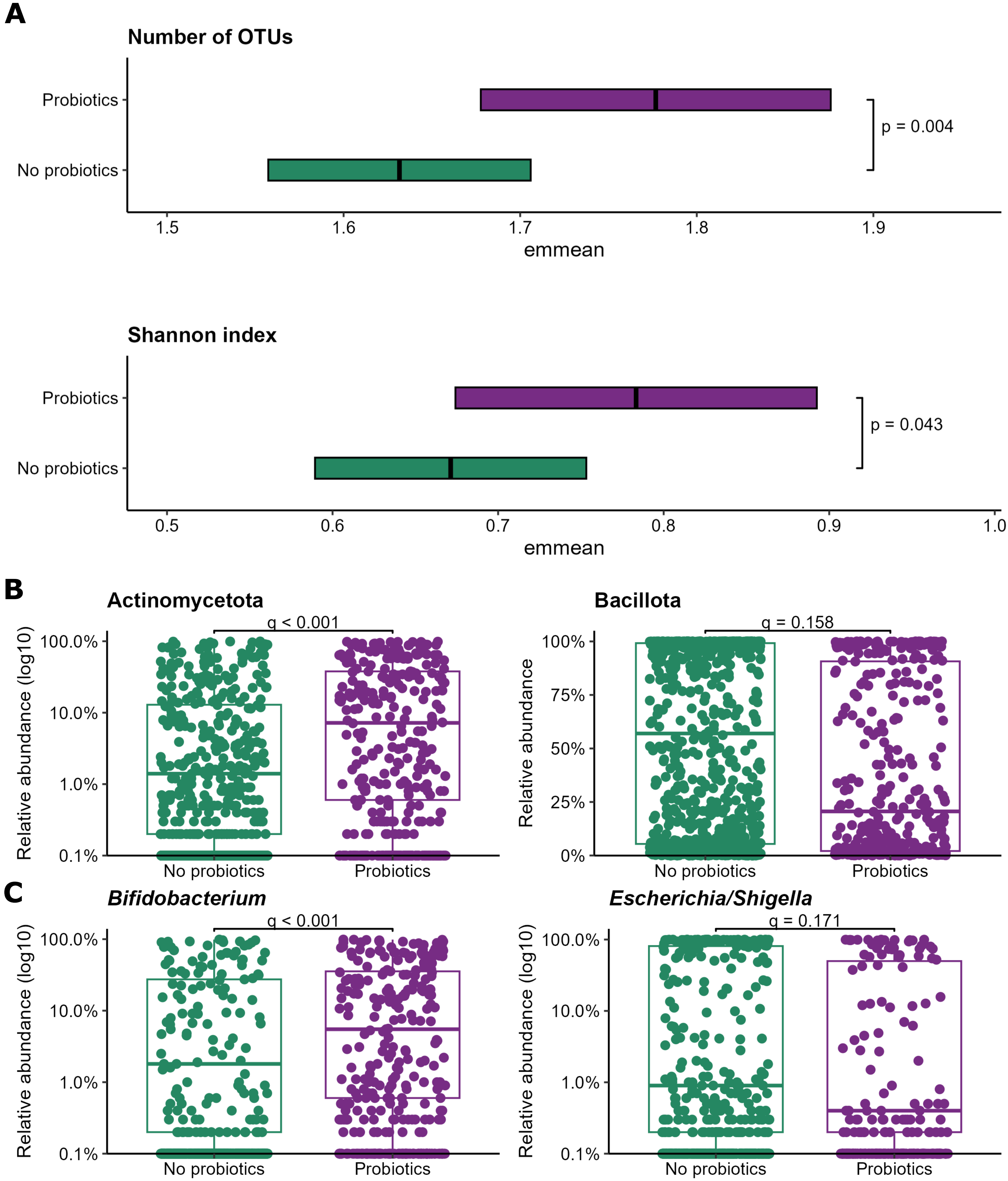
A
Number of OTUs
Probiotics
No probiotics
p = 0.004
1.5
1.6
1.7
1.8
1.9
emmean
Shannon index
Probiotics
No probiotics
p = 0.043
0.5
0.6
0.7
0.8
0.9
1.0
emmean
B
Actinomycetota
q < 0.001
Relative abundance (log10)
100.0%
10.0%
1.0%
0.1%
No probiotics
Probiotics
Bacillota
q = 0.158
Relative abundance
100%
75%
50%
25%
0%
No probiotics
Probiotics
C
Bifidobacterium
q < 0.001
Relative abundance (log10)
100.0%
10.0%
1.0%
0.1%
No probiotics
Probiotics
Escherichia/Shigella
q = 0.171
Relative abundance (log10)
100.0%
10.0%
1.0%
0.1%
No probiotics
Probiotics
