# Supplementary material for: The impact of prophylactic azithromycin on the developing preterm gut microbiome and resistome
Source: Gut Microbes. 2026 Oct 15;18(1):2748147. doi: 10.1080/19490976.2026.2748147 (PMC13679317; doi:10.1080/19490976.2026.2748147)
Supplement: Supplementary file 2 [file KGMI_A_2748147_SM8533.pdf]

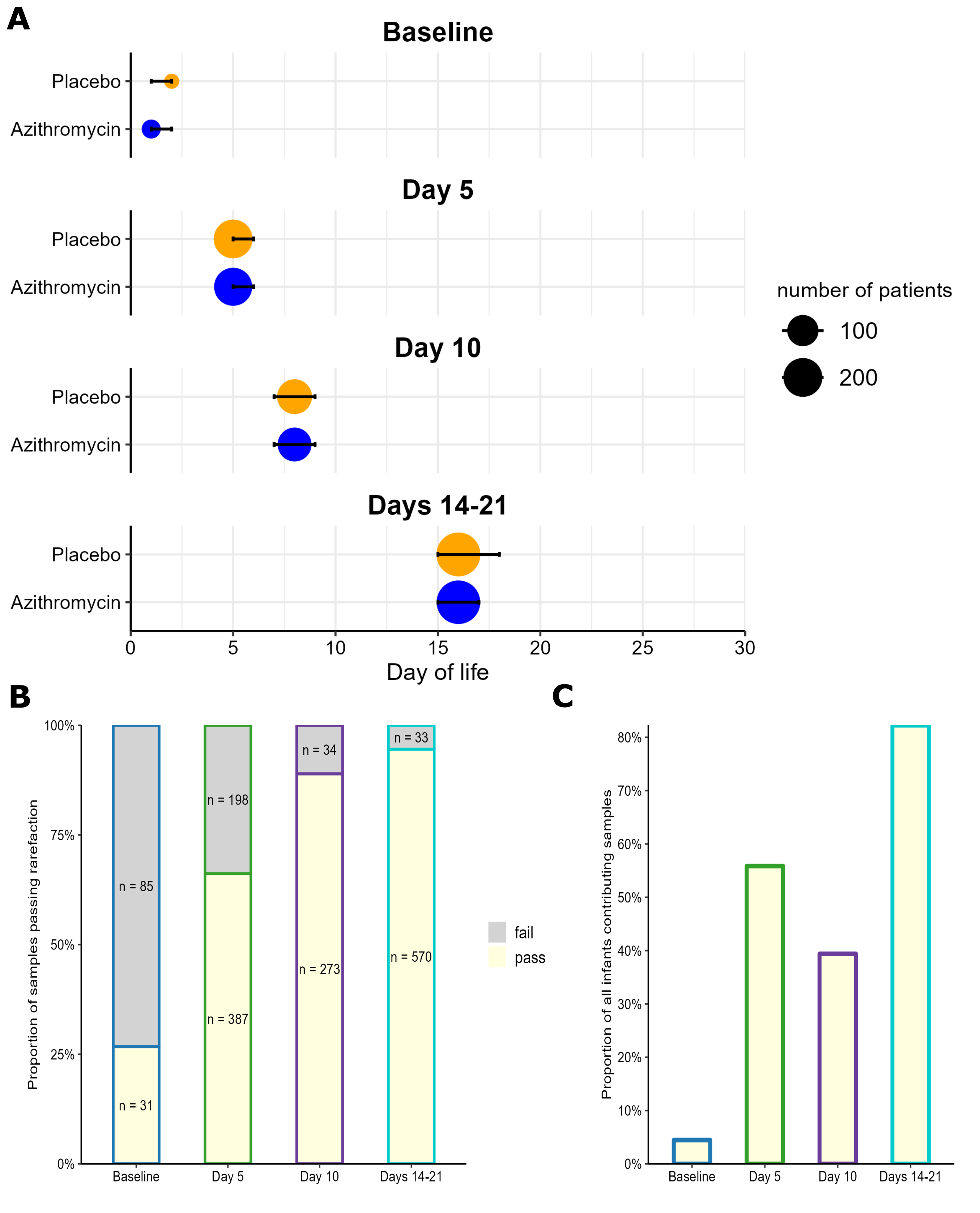
A
Baseline
Placebo
Azithromycin
Day 5
Placebo
Azithromycin
Day 10
Placebo
Azithromycin
Days 14-21
Placebo
Azithromycin
0
5
10
15
20
25
30
Day of life
number of patients
100
200
B
Proportion of samples passing rarefaction
100%
75%
50%
25%
0%
n = 85
n = 31
n = 198
n = 387
n = 34
n = 273
n = 33
n = 570
Baseline
Day 5
Day 10
Days 14-21
fail
pass
C
Proportion of all infants contributing samples
80%
70%
60%
50%
40%
30%
20%
10%
0%
Baseline
Day 5
Day 10
Days 14-21
