# Supplementary material for: The impact of prophylactic azithromycin on the developing preterm gut microbiome and resistome
Source: Gut Microbes. 2026 Oct 15;18(1):2748147. doi: 10.1080/19490976.2026.2748147 (PMC13679317; doi:10.1080/19490976.2026.2748147)
Supplement: Supplementary file 3 [file KGMI_A_2748147_SM8534.pdf]

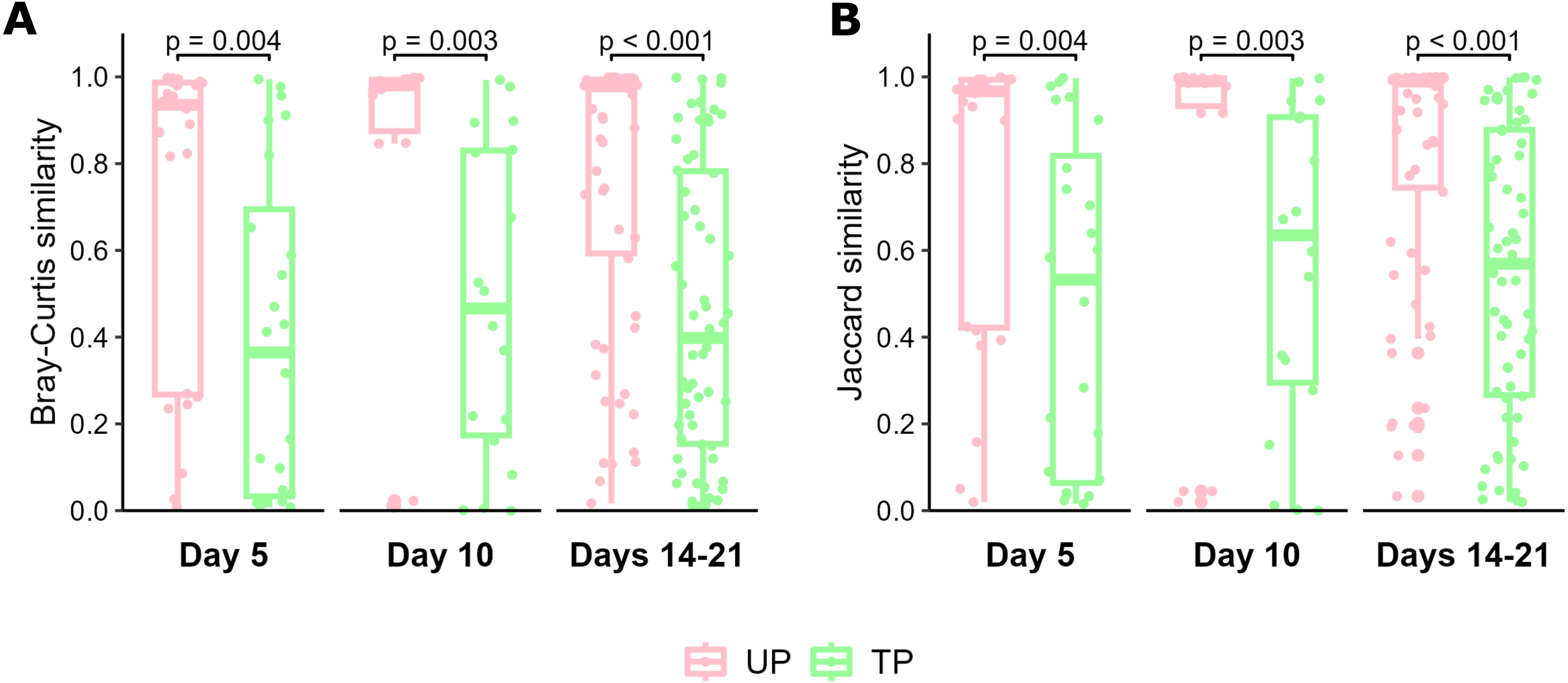
A
B
Bray-Curtis similarity
Jaccard similarity
p = 0.004
p = 0.003
p < 0.001
1.0
0.8
0.6
0.4
0.2
0.0
Day 5
Day 10
Days 14-21
UP
TP
