# Supplementary material for: The impact of prophylactic azithromycin on the developing preterm gut microbiome and resistome
Source: Gut Microbes. 2026 Oct 15;18(1):2748147. doi: 10.1080/19490976.2026.2748147 (PMC13679317; doi:10.1080/19490976.2026.2748147)
Supplement: Supplementary file 4 [file KGMI_A_2748147_SM8535.pdf]

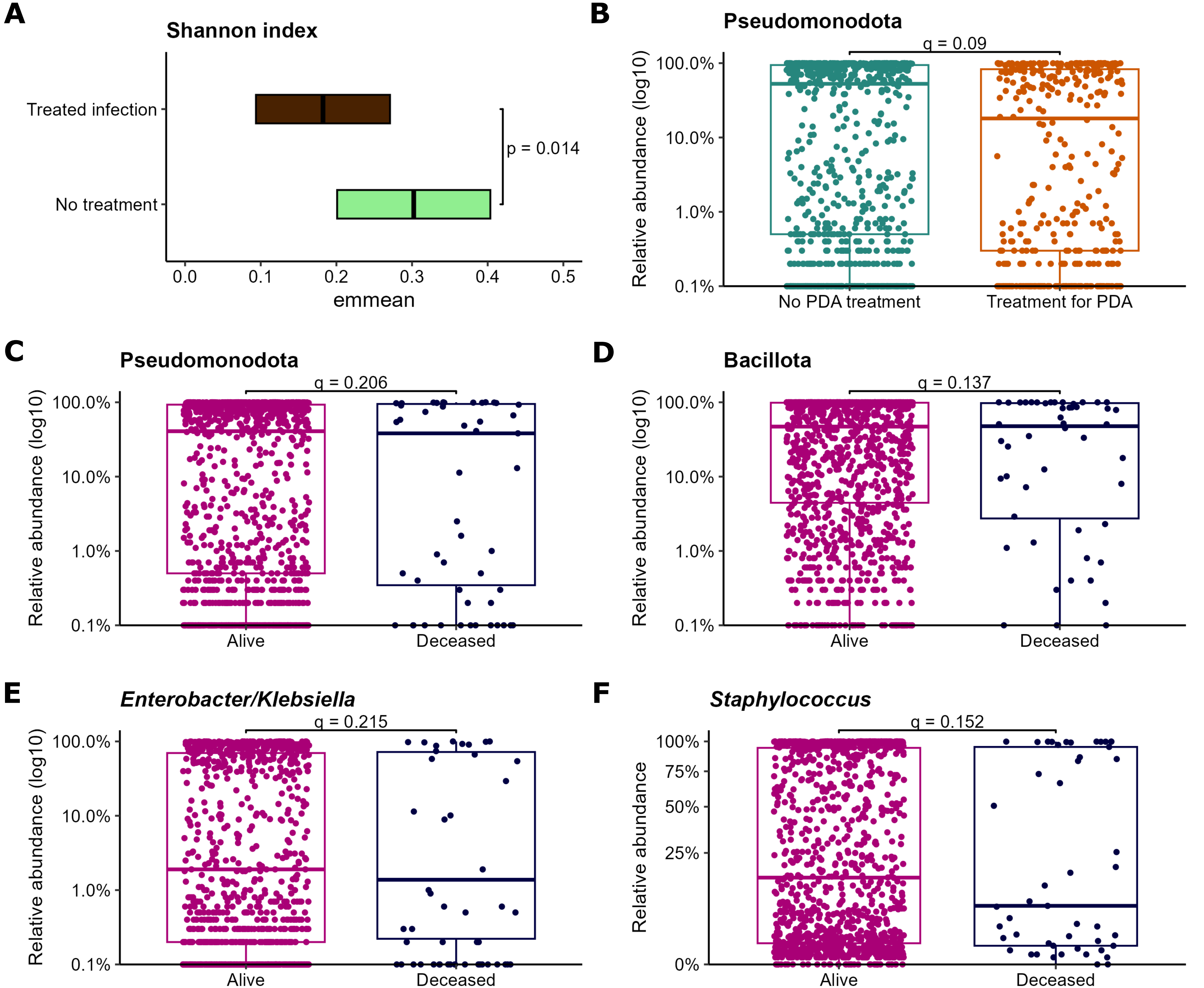

A
Shannon index
Treated infection
No treatment
p = 0.014
0.0
0.1
0.2
0.3
0.4
0.5
emmean
B
Pseudomonodota
q = 0.09
Relative abundance (log10)
100.0%
10.0%
1.0%
0.1%
No PDA treatment
Treatment for PDA
C
Pseudomonodota
q = 0.206
Relative abundance (log10)
100.0%
10.0%
1.0%
0.1%
Alive
Deceased
D
Bacillota
q = 0.137
Relative abundance (log10)
100.0%
10.0%
1.0%
0.1%
Alive
Deceased
E
Enterobacter/Klebsiella
q = 0.215
Relative abundance (log10)
100.0%
10.0%
1.0%
0.1%
Alive
Deceased
F
Staphylococcus
q = 0.152
Relative abundance
100%
75%
50%
25%
0%
Alive
Deceased
