# Supplementary material for: The impact of prophylactic azithromycin on the developing preterm gut microbiome and resistome
Source: Gut Microbes. 2026 Oct 15;18(1):2748147. doi: 10.1080/19490976.2026.2748147 (PMC13679317; doi:10.1080/19490976.2026.2748147)
Supplement: Supplementary file 5 [file KGMI_A_2748147_SM8541.pdf]

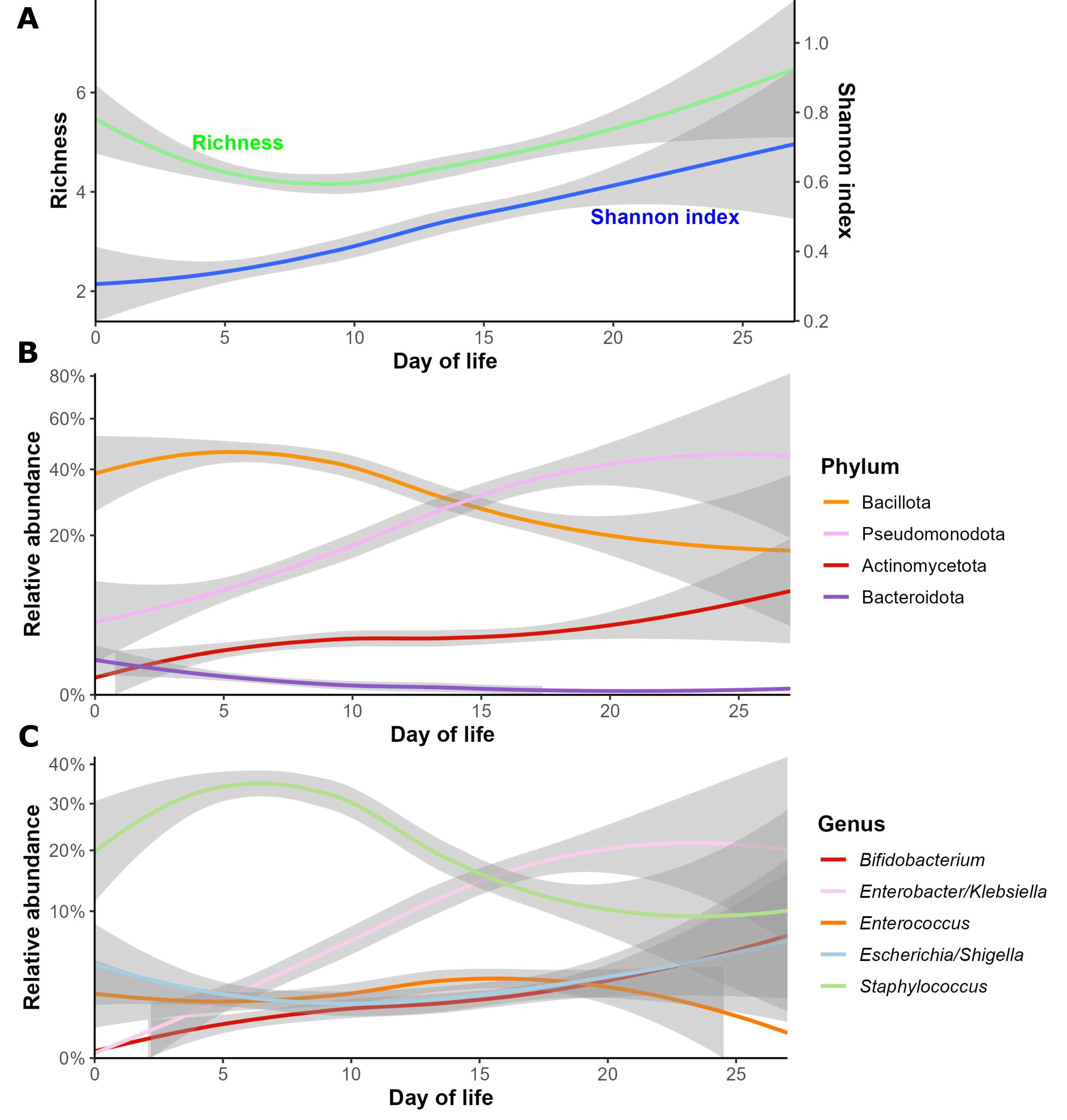

A
Richness
Shannon index
Day of life
B
Relative abundance
Day of life
Phylum
Bacillota
Pseudomonodota
Actinomycetota
Bacteroidota
C
Relative abundance
Day of life
Genus
Bifidobacterium
Enterobacter/Klebsiella
Enterococcus
Escherichia/Shigella
Staphylococcus
