# Supplementary material for: The impact of prophylactic azithromycin on the developing preterm gut microbiome and resistome
Source: Gut Microbes. 2026 Oct 15;18(1):2748147. doi: 10.1080/19490976.2026.2748147 (PMC13679317; doi:10.1080/19490976.2026.2748147)
Supplement: Supplementary file 6 [file KGMI_A_2748147_SM8543.pdf]

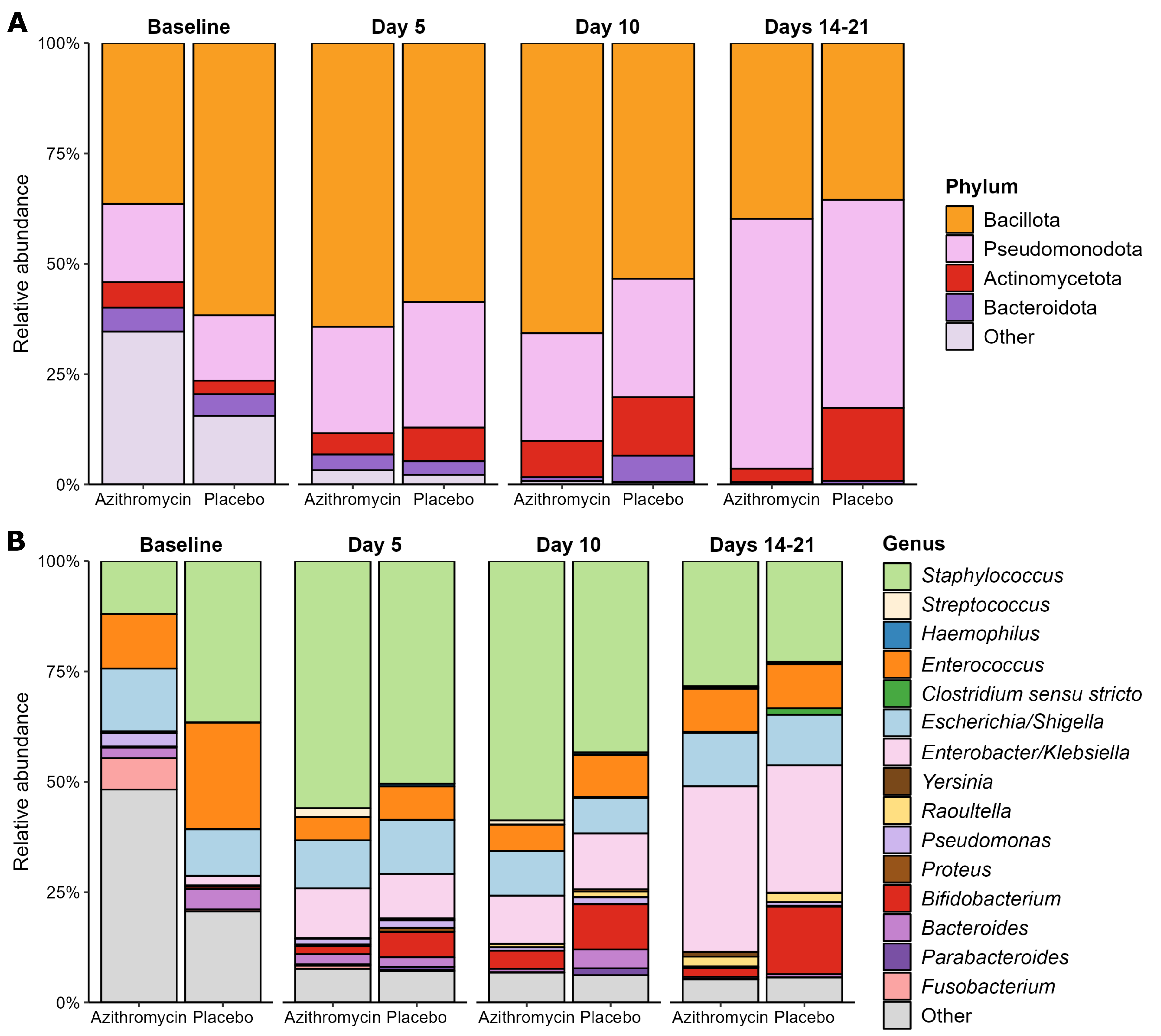
A
Baseline
Day 5
Day 10
Days 14-21
100%
75%
50%
25%
0%
Relative abundance
Azithromycin
Placebo
Phylum
Bacillota
Pseudomonodota
Actinomycetota
Bacteroidota
Other
B
Genus
Staphylococcus
Streptococcus
Haemophilus
Enterococcus
Clostridium sensu stricto
Escherichia/Shigella
Enterobacter/Klebsiella
Yersinia
Raoultella
Pseudomonas
Proteus
Bifidobacterium
Bacteroides
Parabacteroides
Fusobacterium
Other
