# Supplementary material for: The impact of prophylactic azithromycin on the developing preterm gut microbiome and resistome
Source: Gut Microbes. 2026 Oct 15;18(1):2748147. doi: 10.1080/19490976.2026.2748147 (PMC13679317; doi:10.1080/19490976.2026.2748147)
Supplement: Supplementary file 7 [file KGMI_A_2748147_SM7813.docx]

**Supplementary** **Figure 1. Overview of sampling and retention of samples according to time point.** **a)** Day of life (DOL) of patient samples used in the analysis, separated by treatment group (azithromycin or placebo) and time point (baseline, day 5, day 10, or days 14-21). Points represent the median DOL of samples and bars show the interquartile ranges. Points are coloured according to treatment group and sized relative to the number of samples in each group. Bar plots show **b)** the number (n) and proportion (%) of samples that passed/failed rarefaction (threshold of 1,000 reads/sample), and **c)** the proportion of infants from the overall cohort contributing samples after rarefaction, at each time point (baseline, day 5, day 10, or days 14-21).

**Supplementary** **Figure 2. Descriptive overview of the gut microbiome in preterm infants <30 weeks’ gestation at risk of bronchopulmonary dysplasia.** LOESS fits (95% CIs shaded in grey) over time are shown for a) alpha diversity based on richness and Shannon index, b) relative abundances of the top 4 bacterial phyla, and c) relative abundances of the top 5 bacterial genera of 1,261 stool samples.

**Supplementary** **Figure 3. Beta diversity of samples from twin/triplet pairs versus unrelated pairs at given time points.** Paired a) Bray-Curtis and b) Jaccard distances are shown for twin/triplet pairs (TP) or matched unrelated pairs (UP). UPs were matched based on site and gestational age (+/- two days). P values are from FDR corrected Wilcoxon rank sum tests. TP, twin/triplet pair; UP, unrelated pair.

**Supplementary** **Figure 4. Taxonomic compositions of preterm infants treated with azithromycin or placebo at given time points.** Stacked bar charts show the average relative abundances of classified bacterial a) phyla and b) genera, separated by treatment group, at baseline, day 5, day 10, and days 14-21.

**Supplementary** **Figure 5. Impact of probiotics on preterm gut microbiome.** a) Estimated marginal means (EMMs) (± 95% CIs) are shown for the number of OTUs and Shannon diversity in infants exposed or not exposed to probiotics. EMMs were obtained from mixed-effects linear regression models and p value shown is based on Type II Wald Chi-square test on the model. b,c) Boxplots show the relative abundances of b) phyla and c) genera that differed significantly by probiotic exposure. Boxes are coloured according to probiotic use and show median (centre line) and interquartile ranges (IQR) (box limits); whiskers extend ±1.5∗IQR from box’s quartile. Points outside the whiskers represent outliers. Q values (FDR adjusted p values) shown are from MaAsLin2.

**Supplementary** **Figure 6. Association of preterm gut microbiome with clinical outcomes. a**) Estimated marginal means (EMMs) (± 95% CIs) are shown for the Shannon diversity of samples, coloured according to nosocomial infection treatment status (treated infection, or no treatment). EMMs were obtained from mixed-effects linear regression models and p value shown is based on Type II Wald Chi-square test on the model. **b-f)** Boxplots show the relative abundances of **b-d)** phyla and **e,f)** genera that differed significantly according to **b)** patent ductus arteriosus (PDA) treatment status and **c-f)** mortality status. Boxes show median (centre line) and interquartile ranges (IQR) (box limits); whiskers extend ±1.5∗IQR from box’s quartile. Points outside the whiskers represent outliers. Q values (FDR adjusted p values) shown are from MaAsLin2. PDA, patent ductus arteriosus.
